# New information on the Hind limb feathering, soft tissues and skeleton of *Microraptor* (Theropoda: Dromaeosauridae)

**DOI:** 10.1186/s12862-025-02372-0

**Published:** 2025-04-24

**Authors:** Matthieu Chotard, Xiaoli Wang, Xiaoting Zheng, Thomas G. Kaye, Maxime Grosmougin, Luke Barlow, Martin Kundrát, T. Alexander Dececchi, Michael B. Habib, Juned Zariwala, Scott Hartman, Xing Xu, Michael Pittman

**Affiliations:** 1 School of Life Sciences, The Chinese University of Hong Kong, Shatin, Hong Kong SAR China; 2 Foundation for Scientific Advancement, Sierra Vista, Arizona, 85650 United States of America; 3 Institute of Geology and Paleontology, Linyi University, Linyi City, Shandong 276005 China; 4 Shandong Tianyu Museum of Nature, Pingyi, Shandong 273300 China; 5 Center for Integrative Paleobiology, Technology and Innovation Park, Pavol Jozef Šafárik University, Košice, SK-04011 Slovak Republic; 6 Division of Natural Sciences, Dakota State University, Madison, SD United States of America; 7 David Geffen School of Medicine, University of California Los Angeles, Los Angeles, CA United States of America; 8 School of Life and Environmental Sciences, College of Health and Sciences, University of Lincoln, Brayford Pool Campus, Lincoln, UK; 9 Department of Integrative Biology, University of Wisconsin, Madison, WI USA; 10 Centre for Vertebrate Evolutionary Biology, School of Life Sciences, Yunnan University, Chenggong, Kunming, 650504 China; 11 Key Laboratory of Vertebrate Evolution and Human Origins, Institute of Vertebrate Paleontology and Paleoanthropology, Chinese Academy of Sciences, Beijing, 100044 China

**Keywords:** *Microraptor*, Theropod, Paravians, Feathered dinosaur, Anatomy, Leg, Feathering, Flight evolution

## Abstract

**Background:**

*Microraptor* is known as the most significant example of extended feathering on the legs of a paravian, both fossil and modern. Its striking difference with most paravians contributes to the multiple theories on the function of its conspicuous hind limbs. Recent studies tried to uncover its evolutionary significance, but its anatomy has only been described from a small number of samples.

**Results:**

Through the analysis of 16 specimens of *Microraptor*, including 8 previously undescribed specimens, here we provide new information on the structure and number of hindwing feathers within a revised feather taxonomy, including a revised shape of the hindwing *Microraptor* which displays feathers all along the hind limb, except along its pedal digits. Here we describe in detail 6 feather types: metatarsal remiges, long metatarsal coverts, long femoral feathers as well as the first description of long tibial feathers, anterior coverts and minor coverts. Our study of specimens BMNHC PH881 and STM 5–5, 5–75, 6–62 and 6–86 is partially consistent with previous work, but the key difference in this study is a proximal shift of the triangular wing portion formed by the long tibial feathers and the long metatarsal coverts that outlines the joint between the tibiotarsus and metatarsus. This configuration does not exist in any extant or fossil bird, or in any other non-avian paravian described so far, underscoring the uniqueness of *Microraptor*. Unlike previous reconstructions, here the long metatarsal coverts display an asymmetrical close-vanned structure as in the metatarsal remiges. The feathers as preserved are posteriorly projected along the metatarsus and vary between medioposterior and lateroposterior projection along the tibial feathers.

**Conclusions:**

The overall configuration of feather layers is only found in *Microraptor*, and the two layers of elongated and asymmetrically vaned feathers linked to the metatarsus are more reminiscent of the forewing of modern birds than of any leg in other fossils and modern taxa. These new observations allow us to better understand the flight, non-flight locomotion and hunting strategies of this iconic ‘four-winged’ dinosaur suggesting *Microraptor* had a complex behaviour that made it adapted to arboreal and terrestrial habitats.

**Supplementary Information:**

The online version contains supplementary material available at 10.1186/s12862-025-02372-0.

## Background

Most modern birds have a scaly foot called a podotheca that typically extends to the metatarsus [1, 2]. In unscaled portions of the leg, modern birds have hind limb feathers that can be pennaceous, plumulaceous and filamentous. These feathers are comparatively more developed among neognaths than palaeognaths: in neognaths, they can be present along the metatarsus as exemplified by wild and domestic Galliformes such as the light Brahma breed of domestic chicken (*Gallus gallus*) and the Willow Ptarmigan (*Lagopus lagopus*) [1, 3–5], Columbiformes such as grouse-legged breeds of domestic pigeons (*Columba livia*) and birds of prey (Accipitriformes) such as the Rough-legged Hawk (*Buteo lagopus*) [6, 7]. While there has been work on the genetics of hind limb scales and feathering in extant avians [8, 9], there has been little investigation of the anatomy and function of their hind limb feathering.

Pennaceous and non-pennaceous feathers are also found on the legs of early fossil birds, their closest relatives, the non-avian paravians, such as *Anchiornis* [10], *Sapeornis* [11] or *Archaeopteryx* [12]. In early diverging non-avian paravians, the proceratosaurid *Yutyrannus* [13] or the compsognathid *Sinocalliopteryx* [14] also exhibit feathers but they are not pennaceous. Unlike modern birds, the leg feathering of early paravians covered larger portions of the leg and the feathers generally appear to be more elongated, especially the tibial and metatarsal feathers. *Microraptor* appears to have the longest leg feathers relative to body size among all paravians, which are located on the metatarsus [15]. Among early paravians, *Microraptor* has been confirmed to have asymmetrically vaned leg feathers (including its uniquely elongated metatarsal feathers [16, 17]) and vane asymmetry has been suggested in *Archaeopteryx* [18]. We know that the vane asymmetry of forewing feathers is strongly implicated in flight capabilities for modern birds [19, 20]. While the functional impact of vane asymmetry in early paravian leg feathers is less clear, these feathers can be arranged into large surface areas, leading to past suggestions that they were used for flight [4, 17]. We now know there is a diversity in hind limb feathering structure, pattern, extent and size among early paravians [15], including those that were or were not potential flyers (e.g., *Archaeopteryx*, *Anchiornis*,* Jianianhualong*, or *Changyuraptor*), but hind limb feather anatomy and function remains relatively understudied.

The dromaeosaurid microraptorine *Microraptor* is the first and particularly well known ‘four winged’ paravian with proposed flight capabilities [17]. Previously studies supported its gliding behaviour [21, 22] and even powered flight potential [23, 24]. The existing hind limb life reconstruction of *Microraptor* of Li et al. [16] shows between four and five layers of feathers, with a maximum of five layers of metatarsal feathers, but the feathering is not described in detail. According to previous observations, the longest feathers are asymmetrically vaned, curved and linked to the middle of the metatarsus [16, 25]. Also, another layer of shorter feathers outlining the whole hind limb as well as one layer of tibial, a layer of femoral feathers and a layer of anterior feathers were previously described in specimens BMNHC PH881 and IVPP V13352 [16, 17]. The other feathers are not described even though the shortest coverts are drawn on the reconstruction of [16].

Here we report a broad analysis of hind limb feathering in *Microraptor* to fill in key knowledge gaps in our understanding of its anatomy and function. Using this new data, we make extended comparisons between the hind limb feathering of modern birds and iconic early paravians, including *Archaeopteryx* and *Anchiornis*, and use this to re-evaluate what we know about hindwing use during the early evolution of theropod flight. This involved studying more than 1000 early paravian specimens, including ~ 100 specimens of *Microraptor* of which 16 were studied in detail with the aid of Laser-Stimulated Fluorescence to help increase the anatomical information available for study.

## Methods

### Materials

Chosen among hundreds of specimens, the sample of our study includes: *Microraptor zhaoianus* holotype IVPP V12230, *Microraptor gui* holotype IVPP V13352 and referred specimen IVPP V13320 from the Institute of Vertebrate Paleontology and Paleoanthropology, Beijing (IVPP). *Microraptor zhaoianus* specimen BMNHC PH881 has been studied using images from Li et al. [16]. The original specimen is from the Beijing Museum of Natural History, Beijing (BMNHC). Our dataset mostly comprises *Microraptor* sp. specimens STM 5 − 4, 5–5, 5–9, 5–75, 5–93, 5-109, 5-142, 5-150, 5-172, 5-221, 6–86 and 6–62 from the Shandong Tianyu Museum of Nature, Pingyi (STM).The new specimens STM 5 − 4, 5–5, 5–9, 5–75, 5–93, 5-109, 5-142, 5-150, 5-172, 5-221, 6–62 and 6–86 were all assigned to the subfamily Microraptorinae based on the presence of anatomical characteristics used in past studies [25–32] (Additional file 1: Table S1): the caudal chevrons have very elongated posterior extensions; large supracoracoid fenestra; narrow-waisted coracoid; semilunate distal carpal that is small and covers about half the base of metacarpals I and II; dorsally situated articular surface on manus unguals; combined length of metacarpal I plus phalanx I-1 is equal to or less than the length of metacarpal II; pelvis opisthopubic with boot bent sharply backwards; prominent lateral tubercle on the pubic shaft; nearly vertical ischium with enlarged obturator process; subarctometatarsalian pes; femur with elevated head (above greater trochanter); metatarsal V over 50% length of metatarsal IV; metatarsal I more distally situated than other known dromaeosaurids. These specimens were assigned to *Microraptor* based on characters [17, 27, 32, 33] (Additional file 1: Table S1): skull lacks surface ornamentation of *Sinornithosaurus* as well as subfenestral fossa; the manual phalanx III-3 is extremely slender, the dorsoventral thickness of its midshaft is about 1/3 to 1/2 lateral width of phalanx III-1; the length ratio of manual phalanx III-1 to II-1 is close to 0.8; manual III-3 extremely slender and shorter than III-1; extremely short manual phalanx III-2 that is less than one quarter of the length of manual III-1; small distal articulation of manual III-3 skewed ventrally; second sacral centrum is transversely widened, ratio of bilateral width between second sacral centrum and last dorsal centrum is about 2.60; ischium is slender in lateral view with anteroposterior width of distal end about 40% of the proximodistal length along the posterior margin; very strongly recurved and slender pedal unguals with prominent flexor tubercles. Therefore, we adopt the approach of Wang and Pei [32] to assign specimens to the genus level only because of the controversy surrounding the diagnoses and interrelationships of *M. zhaoianus*, *M. gui*, and *M. hanqingi* which are potentially all synonymous [25, 26, 32, 34, 35]. A sample of the mid-diaphysis of the femur and tibia was extracted from *Microraptor* specimen D-2842 (Dalian Natural History Museum, Liaoning, China) to provide osteohistological information about the growth of the stylopodium and zeugopodium. D-2842 is an almost complete but not well-preserved specimen with a left femur length of 86 mm.

The modern bird specimens observed for their hind limb feathers are represented by 157 naturalised specimens from 23 orders. They are stored in three different institutions: the Museo Argentino de Ciencias Naturales, Buenos Aires (MACN), the Hong Kong Biodiversity Museum, Hong Kong (HKBM) and the University of Michigan Museum of Zoology, Ann Arbor (UMMZ). For further details, see electronic supplementary material (see Additional file 1: Table S2).

### Laser-stimulated fluorescence

Each specimen was examined using white light (WL) and Laser-Stimulated Fluorescence (LSF), apart from specimen BMNHC PH881 which was only observed under WL. LSF imaging is based on the procedure described in Kaye et al. [36]. A 0.5 W 405-nm laser diode was used to fluoresce the fossil specimens according to standard laser safety protocol. Thirty-second time-exposed images were taken with a Nikon D810 DSLR camera fitted with a 425-nm laser-blocking filter. The postprocessing was conducted consistently on the full set of photos (equalisation, saturation, and colour balance) in graphics software Adobe Photoshop CS6. This allowed the information captioned by the camera to be fully represented and for specific areas of interest to be seen as clearly as possible.

### Histological analyses

The mid-shaft samples were scanned on the bending magnet beamline 20B2 of the SPring-8 facility of Japan Synchrotron Radiation Research Institute (JASRI; [37]) with an effective isotropic voxel size of 2.75 μm and a monochromatic beam set at an energy of 30 keV. The micro-CT system consists of high-precision stages and an X-ray image detector, except with the light source and monochromator in the experimental hutch 1 of BL20B2 [38]. We used the propagation phase contrast effect to increase the contrast and visibility of delicate histological features. The propagation distance was 200 mm from the specimen to the X-ray image detector; 3600 projections were collected for each bone sample, and the exposure time for each projection was 150 msec. All scans were analysed using VGStudio Max 3.0. (Volume Graphics, Heidelberg, Germany). The images were processed using CorelDRAW X5 software. Histological measurements were taken from digitised cross-sections using ImageJ software.

### Comparative methods

Measurements of length and angles were made from photos using Adobe Photoshop 2019 software. The figures were produced with Adobe Illustrator 2019 software. All ratios were calculated using the software Microsoft Excel.

We used standard comparative anatomy methods to compare our studied specimens with closely related early diverging paravians such as *Anchiornis*, *Archaeopteryx* and *Changyuraptor*. We also compared the *Microraptor* specimens with modern bird legs, especially their feathering and soft tissues.

Feather nomenclature was determined according to previous literature and their position, without making a statement of homology with the forewing. We named the shortest pennaceous feathers as minor coverts and the patches on the anterior sideas anterior coverts. We decided not to include corresponding bone names for these two types of feathers because it is harder to identify them individually compared to longer feather types e.g. long metatarsal coverts and metatarsal remiges. Projections of feathers from *Microraptor* bones were measured using the proximal end as the 0° reference and the distal end as the 180° reference.

Pedal ungual measurements taken using the outer angle of the claw bones and sheaths follow the method of Pike and Maitland [39].

We did not consider estimated measurements in our ratio calculations. Feathers were measured from the bone as preserved. Feathers can be taphonomically deformed [40], which can lead to wrong measurements and calculations. In order to avoid these issues and to maximise data quality, we considered a broad sample of feathers where the contours and/or structure is preserved for each specimen studied.

Body mass of 13 *Microraptor* specimens were calculated (see Additional file 1: Table S3) according to the proxy methods of Benson et al. [41] and Campione et al. [42]. Thus, to calculate the body mass, the femoral length has been used to estimate the minimum circumference around the femoral shaft. Although the mass of four published specimens changed from previous studies [26, 43, 44], this uniform method gives mass estimates that allow us to better understand the potential for intrageneric variation among *Microraptor* specimens in this study, which is appropriate given the comparisons we seek to make.

## Results

### Feather distribution and variations between leg segments

**Fig. 1:**
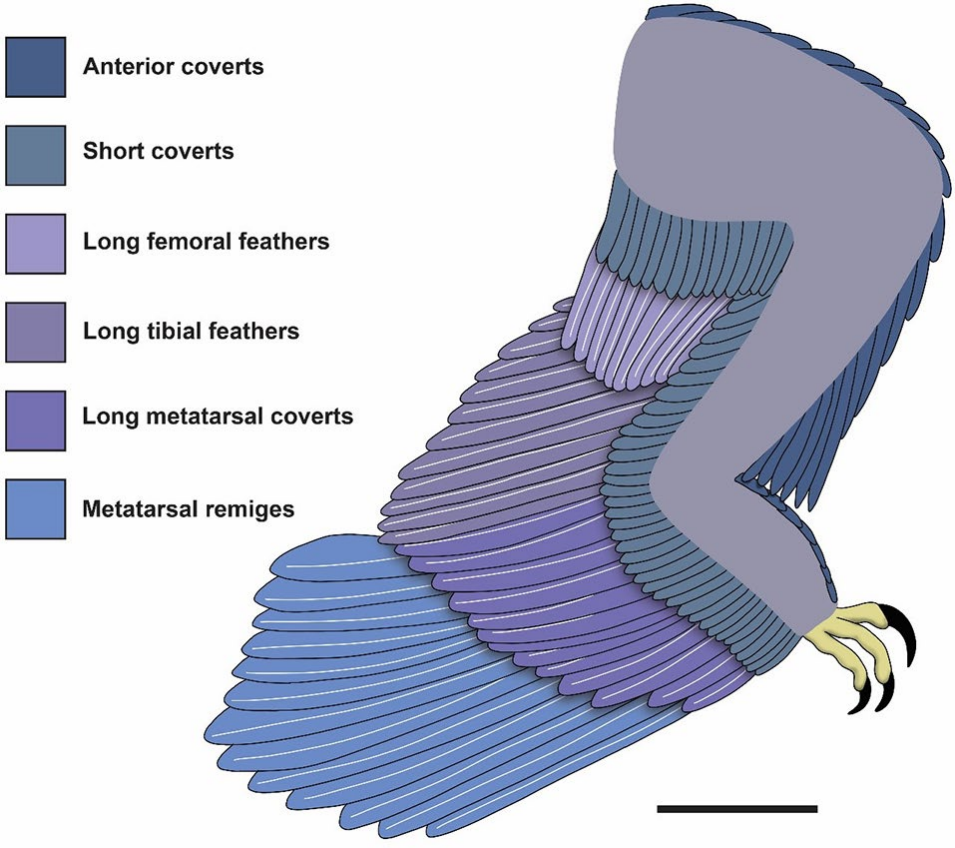
Leg feathering of *Microraptor* using specimens BMNHC PH881 and STM 5–5, 5–75 and 6–86. This reconstruction shows the newly described shape of the hindwing of *Microraptor*. It shows the structure and arrangement of feathers as well as the number of each feather type as observed or estimated using the best-preserved specimens within our sample. Scale bar is 50 mm

Pennaceous feathers are preserved along the entire posterior side of the hind limb of *Microraptor* except the pedal digits of specimens STM 5–5 and 6–86, despite multiple specimens preserving articulated feet including exceptional toe pads [2]. Using all the specimens in our sample, we were able to reconstruct the leg feathering of *Microraptor* (Fig. 1, Additional file 1: Table S4).

Previously, posterior metatarsal feathers have been observed in the microraptorines *Wulong* and *Changyuraptor* [28, 31] and in the anchiornithines *Anchiornis*, *Pedopenna*,* Caihong* and *Serikornis* [45–48]. As preserved on specimens of *Microraptor*, there are 3 layers along the posterior side of the metatarsus. These feathers are differentiated with the longest inferred as metatarsal remiges, mid-sized ones as long metatarsal coverts and the smallest ones as minor coverts. There is also at least one layer of covert feathers on the anterior side which is preserved as a patch. Metatarsal feathers are well-preserved in specimens BMNHC PH881, IVPP V13352 as well as STM 5–5, 5–75, 6–62 and 6–86 and they are likely posteriorly projecting from the metatarsus as seen in STM 6–62. To describe these feathers, we considered these specimens as well as other specimens such as STM 5–9, 5-142 and 5-172 that display relevant details on select feathers.

In previous studies, tibial feathers are described in multiple early paravians specimens such as *Sapeornis* specimen STM 16–18 [4], *Changyuraptor* specimen HG B016 [24], *Wulong* specimen DNHM D2933 [31], *Anchiornis* specimens LPM-B00169 [4], YFGP-T5199 [10] and BMNHC PH804 [47], *Caihong* specimen PMoL-B00175 [45], *Eosinopteryx* specimen YFGP-T5197 [49] as well as the Berlin [18, 50, 51] and Altmühl (11th ) specimen of *Archaeopteryx* [12]. Tibial feathers are inferred as crural feathers in Zheng et al. [4] but we chose to keep the name tibial feathers to conserve the link between these feathers and the tibia. We found that *Microraptor* had two layers of tibial feathers on its posterior side with the longest called long tibial feathers and the shortest called minor coverts. These layers are preserved projecting medioposteriorly in STM 5–5 and lateroposteriorly in STM 5–9 so their mediolateral position appears uncertain. Pennaceous feathers appear to be linked to the tibiotarsus of 12 specimens of *Microraptor* (IVPP V13320 and V13352; STM 5 − 4, 5–5, 5–9, 5–75, 5-109, 5-142, 5-172, 5-221, 6–62 and 6–86), with the tibial feathers best represented in specimens STM 5–5 and 6–86.

Feathers have previously been described along the posterior femur microraptorine *Wulong* [31]. They have also been described in the anchiornithines *Eosinopteryx* and *Serikornis* [47, 49]. In the anchiornithine *Xiaotingia* described by Xu et al. [52], there are potential posterior femoral pennaceous feathers along the half proximal part of the femur with a length of 55 mm, but they are too poorly preserved to distinguish their structure, even though potential rachises are present [52]. In the anchiornithine *Caihong*, pennaceous feathers are also likely present along the posterior surface of the femur, but this was not detailed by Hu et al. [45] and not observed first-hand. Here, by mostly using the specimen STM 6–86 (Fig. 4), we describe the femoral feathers in detail for the first time, reporting two posterior layers called long femoral feathers and short coverts respectively as well as an anterior layer called anterior coverts. In our study, feather rachises are found anchored in the femoral soft tissues of specimens STM 5 − 4 where they are likely close to the bone and 6–86 where the rachises are partially preserved in the soft tissues (Fig. 4). Thus, their attachment point to the femur remains unclear. We also consider the same feathers as attached in BMNHC PH881, IVPP V12330 and V13320 as well as STM 5–5. Despite the new information provided, more myological work as well as the discovery of specimens with even better-preserved feathering would be needed to understand how these feathers were implanted in detail. These feathers were previously described and measured as 1.7 times the length of the femur according to Li et al. [16], but the variation in feather length is unclear and, in their study, it is not specified if they form a straight edge. Here, we observed that these feathers seem to increase in size distally according to our observation of specimen STM 6–86. However, the general pattern remains hypothetical because the most distally situated femoral feathers are obscured by feathers linked to the tibiotarsus. Symmetrically close-vaned feathers associated with the whole posterior femur have been described in the controversial *Microraptor* specimen LPM-0200 [16] previously referred to as BPM 1 3–13 in Norell et al. [53]. It is also unclear how these feathers were implanted to the thigh and future myological work and new discoveries with better preserved rachises anchored in soft tissues will also help to address this.

## Metatarsal feathering

### Metatarsal remiges

**Fig. 2:**
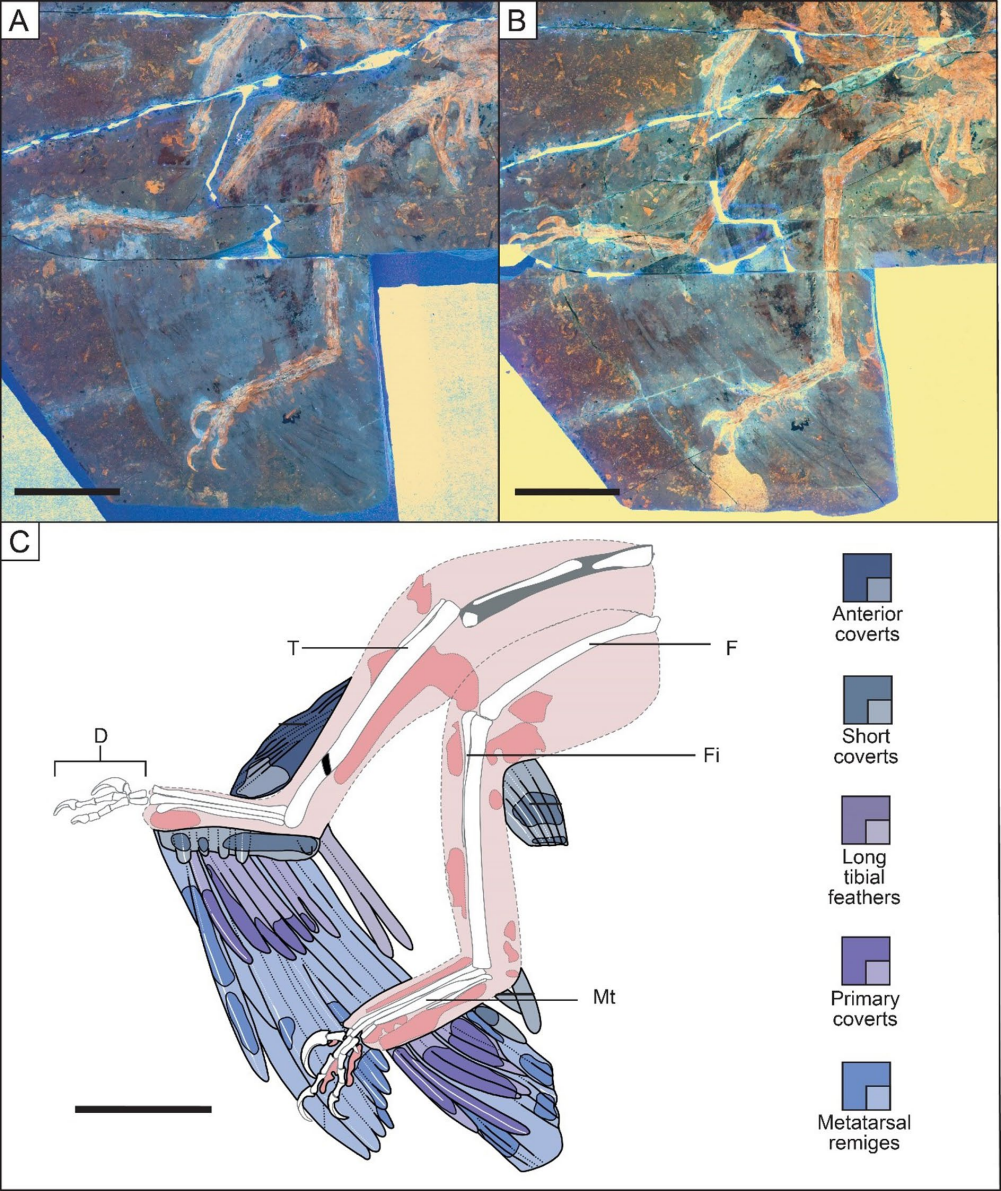
Hind limbs of *Microraptor* specimen STM 5–75 showing the overall shape of the metatarsal feathers. Main slab of specimen STM 5–75 under LSF (**A**). Counter slab of specimen STM 5–75 under LSF (**B**). Reconstructed anatomical line drawing (**C**). Colour coding used in Figs. 2, 3, 4 and 5 is: darker blues/purples, preserved feathers; pale blues/purples, reconstructed feather outlines; white, preserved skeleton; grey, reconstructed skeletal sections; dark pink, preserved soft tissues; pale pink, reconstructed soft tissues. Long-dashed lines are reconstructed outlines; short-dashed lines are breaks between preserved material and reconstructed outlines. D, right digits; F, left femur; Fi, left fibula; Mt, left metatarsus; T, right tibiotarsus. The specimen displays the best preservation of the overall shape of metatarsal remiges with long metatarsal coverts preserved with their barbes. The preservation of some metatarsal remiges and coverts is sufficient to see their degree of asymmetry. Scale bar is 50 mm

**Fig. 3:**
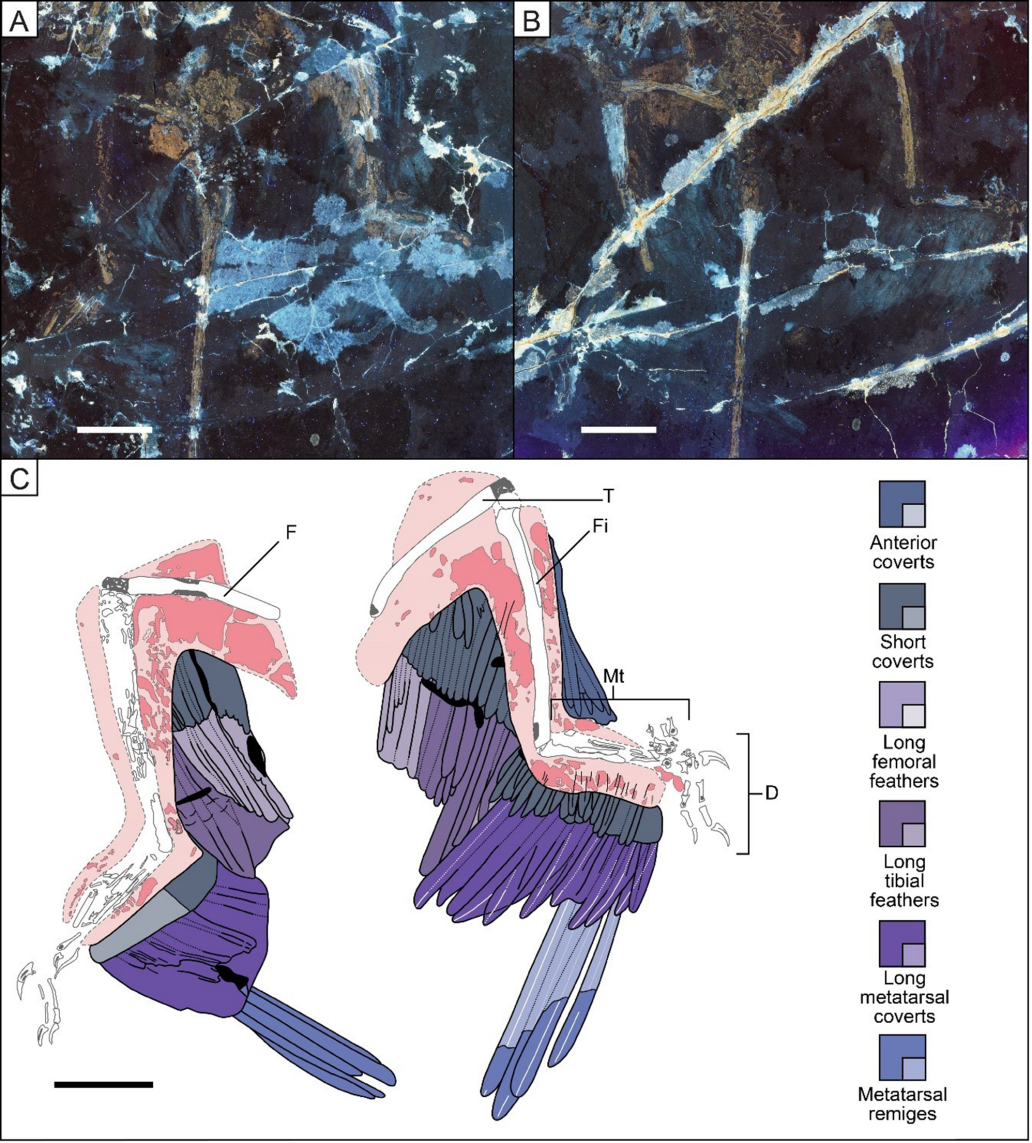
Hind limbs of *Microraptor* specimen STM 5–5 showing the shape of the metatarsal coverts. LSF image ofthe main slab (**A**) and counter slab (**B**). Reconstructed anatomical line drawing (**C**). D, left digits; F, right femur; Fi, left fibula; Mt, left metatarsus; T, left tibiotarsus. The specimen displays the best preservation of the shape of long and short coverts of the metatarsus. Scale bar is 50 mm

**Fig. 4:**
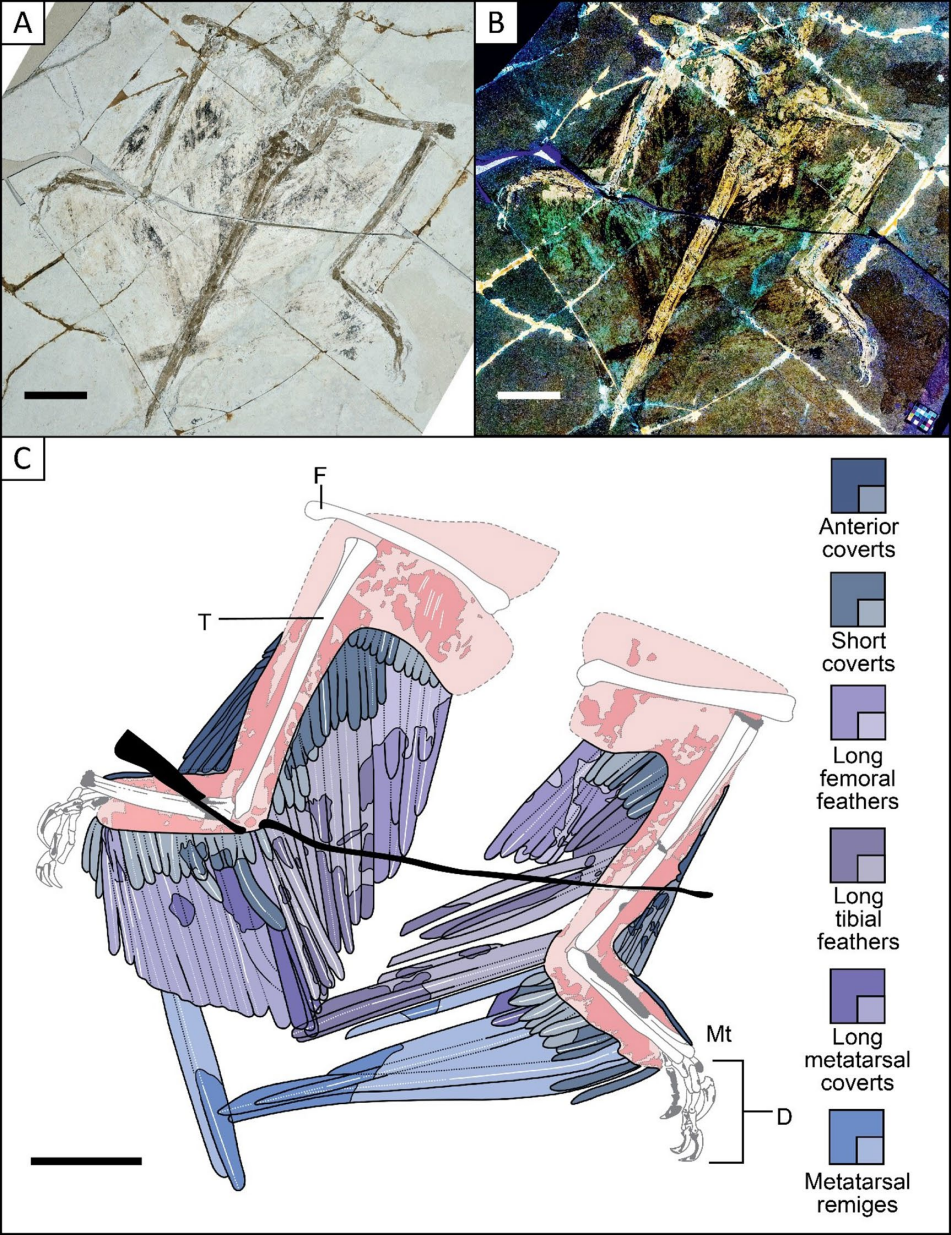
Hind limbs of *Microraptor* specimen STM 6–86 preserve excellent feathering, including tibial and femoral feathers and metatarsal remiges of the wing tip. Photo under white light (**A**) under LSF (**B**) and a reconstructed anatomical line drawing (**C**). D, right digits; F, left femur; Mt, right metatarsus; T, left tibiotarsus. The specimen preserves the best leg feathers overall with especially well-preserved tibial and femoral feathers and the tips of a few metatarsal remiges. Scale bar is 50 mm

**Fig. 5:**
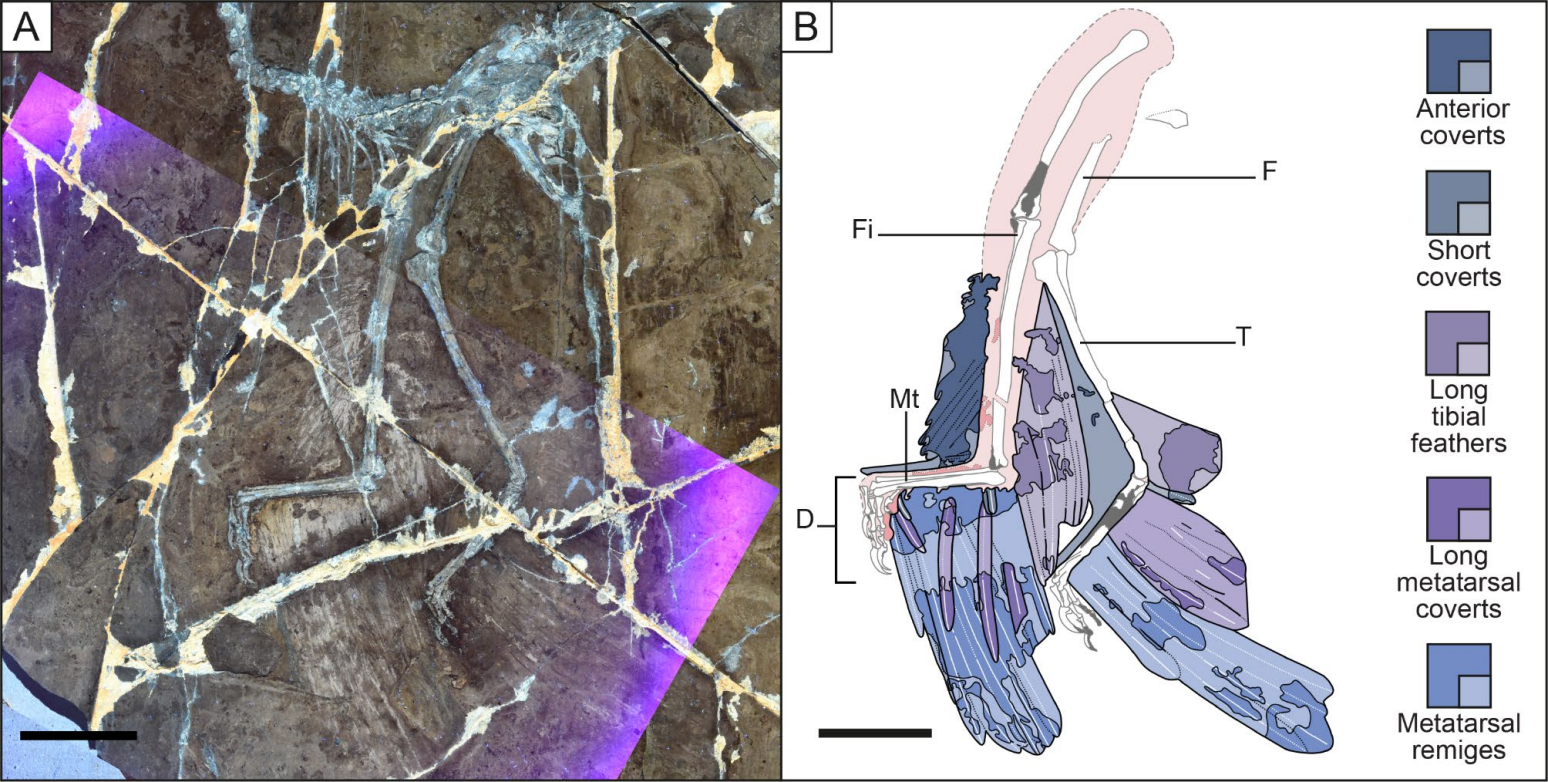
Hind limbs of *Microraptor* specimen STM 6–62 showing well-preserved rachises. Photo under LSF (**A**) and reconstructed anatomical line drawing (**B**). D, left digits; F, right femur; Fi, left fibula; Mt, left metatarsus; T, right tibiotarsus. The specimen displays the best rachis preservation and where they are linked to the bone. Scale represents 50 mm

Metatarsal remiges are linked to the metatarsus and are best preserved in STM 5–75 (Fig. 2) but we can also find them in specimens BMNHC PH881, IVPP V13352 and STM 5–5, 6–86 and 6–62 (see Additional file 2: Figs. S1, S2; Figs. 3, 4 and 5). The metatarsal remex count is likely between 10 (STM 5–75) to 13/14 (IVPP V13352) in specimens where they can be estimated. Other specimens such as STM 5–5 and 6–86 do not preserve enough remiges to estimate their number., Thus, based on the sample we have, we cannot determine if there is any correlation between the number of metatarsal remiges and specimens size. These feathers resemble the primary remiges of the forewing, they are asymmetrically close-vaned as previously described by Xu et al. [17] and Xu [15], and this is most evident in specimen BMNHC PH881 (Fig. S1). They also curve anteriorly as seen in specimen STM 6–62 (Fig. 5). These feathers have small trailing vane barb angles as measured in specimen IVPP V13352 (~ 10–15°; also in Feo et al. [19]). However, a few metatarsal remiges in STM 5 − 4 display a structure comparable to that of the open-vaned feather described in specimen BMNHC PH828 of *Anchiornis* by Saitta et al. [54], but this is probably a preservational artifact. Feather iridescence comes from barbule microstructure according to Li et al. [16]. In their study, *Microraptor* is inferred to have had iridescent feathers based on nanostructures found in preserved barbules. This observation is rare in the fossil record, probably due to the fine size of barbules and their nanostructures [6]. Even in modern bird feathers, barbules are often degraded [55]. In the holotype specimen of *M. gui* IVPP V13352, Xu et al. [17] noted that the longest metatarsal remex was about twice the length of the femur. In this study, a similar ratio is observed in new specimens STM 5–5 and 6–86 (1.92 and 2.04 respectively; femur length of 91.99 mm and 99.05 mm respectively) which have estimated mass of 1.11 kg and 1.62 kg respectively. However, this ratio is higher in specimen BMNHC PH881 (2.32; femur length of 52.51 mm) but this specimen is significantly smaller than the other specimens mentioned, thus raising the possibility of negative allometric growth for hind limb feathering. As there is little difference in this ratio for specimens whose femur length ranges from 86.00 (STM 5–5) − 99.05 mm (STM 6–86), this suggests that either this value stabilises to become isometric later in ontogeny or has another source of variation influencing it beyond body mass. Regardless, relative size seems not to affect the shape of the hindwing, suggesting a continuation of function across the size classes documented here.

By combining observations made in STM 6–86 (Fig. 4), along with specimens BMNHC PH881 and STM 6–62, 5–5 and 5–75 (see Additional file 2: Fig. S1 and Figs. 2, 3 and 5), the shape formed by the longest metatarsal feathers can be reconstructed as close to an isosceles triangle (Fig. 1). The metatarsus serves as its base, the longest metatarsal remiges linked to the middle of it and the edges of the triangle formed by the tips of the remaining feathers with the ventral edge appearing more convexly curved (Fig. 1), relatively similar to the reconstruction in Li et al. [16]. In IVPP V13352 (see Additional file 2: Fig. S2), feather lengths are highly variable making it difficult to differentiate both hindwings. However, the proximal part of the left hindwing seems to have remiges that decrease in size proximally, as observed in specimens BMNHC PH881 and STM 6–62, 6–86, 5–5 and 5–75. Note that on specimen IVPP V13352, the hindwing feathers are slightly displaced across a crack in the slab that is accounted in our reconstruction (Fig. 1) and preservation figure (Additional file 2: Fig. S2). The angle that the remiges attach to the metatarsus varies between ~ 40° on the proximal end for the incomplete feathers in specimen STM 5-142 (see Additional file 2: Fig. S3) and ~ 110° on the distal end in specimen STM 6–62 where the rachises display a curve (Fig. 5). In specimen BMNHC PH881 the remiges on the proximal end attach at a more acute angle of ~ 65°, whereas the remiges on the distal end attach at an angle of ~ 70°. In early paravians and modern birds there is no equivalent of long asymmetrically vaned feathers along the metatarsus. Nevertheless, it is possible to compare the angle with the longest feathers along the metatarsus of specimen LPM-B00169 of *Anchiornis*. In that specimen, the longest feathers display an angle of ~ 135° relative to the whole left metatarsus and it varies along the right metatarsus with an angle of ~ 90° along the whole bone. The ratio of the longest metatarsal remex to the femur is approximately 0.72 according to Fig. 2 of Hu et al. [46].

### Long metatarsal coverts

Metatarsal remiges are overlain by shorter feathers that also attach to the metatarsus and these feathers are inferred as long metatarsal coverts, in keeping with the naming conventions for modern bird forewing. Long metatarsal coverts have previously been mentioned by Li et al. [16] in BMNHC PH881, but they have not been described in detail for *Microraptor* until now. The number of these feathers varies between 12 and 14, as preserved in new specimens STM 5-142 and 5–5 respectively (see Additional file 2: Fig. S3; Fig. 3). These feathers have closed and asymmetrical vanes and a smooth posteriorly curved shape, as observed in new specimens STM 5–5 and 5-172 and potentially in STM 5–75 as well (see Fig. 2 and Additional file 2: Fig. S4; Fig. 3). The long metatarsal covert length to metatarsal remex length ratio was calculated as 0.51 with the longest metatarsal remex associated with a long metatarsal covert on the mid-section of the metatarsus of specimen STM 5–5 (Fig. 3). A ratio of 0.69 was also calculated using a proximal metatarsal remex and its associated long metatarsal covert for specimen IVPP V13320 (see Additional file 2: Fig. S5). It was not possible to calculate this ratio in other specimens because they lacked the appropriate feather preservation necessary to do so. The feathering pattern of the long metatarsal coverts varies considerably across the studied specimens. The major morphs identified show an increase in size proximally in specimens BMNHC PH881 and STM 5–5, 6–62 and 6–86 (see Additional file 2: Fig. S1; Figs. 3, 4 and 5), but remain uniform in length in specimens STM 5-142 and 5–75 (see Additional file 2: Fig. S3; Fig. 2). These feathers also vary in projection angle relative to the metatarsus. The long metatarsal coverts can be more inclined relative to the proximal end of the bone with an angle of ~ 40° and they can become less inclined to reach an angle of ~ 60° on the distal end, as seen in specimen STM 5-142 (see Additional file 2: Fig. S3). These feathers can also be more inclined relative to the distal end of the bone with an obtuse angle between ~ 110° and ~ 135°, as seen in STM 5-172 (see Additional file 2: Fig. S4), with the preservation of their rachises only on the distal end of the metatarsus.

### Minor coverts

In Zheng et al. [4], short pennaceous feathers along the posterior metatarsus appear to be reconstructed in the early paravian *Anchiornis* based on LPM B00169 and in the early pygostylian *Sapeornis* based on specimen STM 16–18. Similar feathers are described here for *Microraptor*, which vary in number from at least 14 based on specimen STM 5–9 (missing a few feathers on the proximal end) to around 20 based on specimen STM 5–5 where this feathering is almost perfectly preserved (see Additional file 2: Fig. S6; Fig. 3). The angle between the feathers and the bone is ~ 90° in STM 6–62 by considering the feathers preserved on the distal left metatarsus and on its proximal portion (Fig. 5). However, by considering the partially preserved feather with a rachis on the proximal end of the right metatarsus, the angle is ~ 70° relative to the bone. In specimen STM 5–5 (Fig. 3), the short coverts vary in angle between ~ 55° and ~ 80° along the metatarsus and on its proximal end, around the condyle, the angle is up to ~ 150°. These feathers are relatively similar in size along the bone in specimens STM 5–5 and 5–9 where they are best preserved (see Additional file 2: Fig. S6; Fig. 3).

#### Anterior coverts

Along the anterior metatarsus, patches of feathers are mostly preserved in specimens STM 5-142 and 6–86 that appear to be plumulaceous downy feathers that follow the shape of the soft tissues and seem to become shorter distally (see Additional file 2: Fig. S3; Fig. 4). However, it was not possible to count them or to measure their angle to the bone.

### Tibiotarsus feathering

#### Long tibial feathers

The long tibial feathers in specimen STM 5-172 appear to be linked to the posterior side of the tibiotarsus and they are less asymmetrically vaned than the long metatarsal coverts (see Additional file 2: Fig. S4), but we were unable to determine whether they were symmetrical or weakly asymmetrically close-vaned. These feathers are mainly obscured in other non-avialan dinosaurs, which makes it difficult to understand their general patterns [4]. The feathers appear straight in specimens IVPP V13352, V13320, STM 5-142, 5–75, 5–5 (see Additional file 2: Figs. S2, S5, S3; Figs. 2 and 3), except in specimens STM 5-172,5 − 4, 5-109 and 6–62, and potentially in STM 6–86, where they are curved distally (see Additional file 2: Figs. S4, S6, S9; Figs. 4 and 5). There is a minimum of 6 long tibial feathers along the tibiotarsus of specimen STM 5–5 and at least 7 in STM 6–86 (Figs. 3 and 4), but this count could be higher as some of these feathers might be obscured by long metatarsal coverts and long femoral feathers. A long tibial feather count of ~ 12 can be estimated according to the size and position of the individual in specimen STM 5-172 (see Additional file 2: Fig. S4). The length of these feathers does not vary a lot along the proximal half of the tibia of STM 5–5 (Fig. 3), but in STM 5-172, these feathers decrease in size in a proximal direction. The projection angle of the tibial feathers relative to the proximal tibiotarsus varies between ~ 160° in specimen STM 5 − 4 and ~ 110° in specimen STM 5-142 and 5-172 (see Additional file 2: Figs. S3, S4, S7). This contrasts with *Sapeornis* (STM 16–18) where the same feathers project from the proximal end of the tibia with an angle of ~ 110° up to an angle of ~ 170° with the most distal portion (personal observation in Zheng et al. [4]). In this pennaraptoran, these feathers curve posteriorly as we observe in *Microraptor* specimens STM 5-172, 5 − 4 and 6–86 (see Additional file 2: Figs. S4, S7; Fig. 4) [4, 56]. Among microraptorines, *Wulong* (DNHM D2933) has straight feathers projecting with obtuse angles along its whole tibiotarsus (observed in Fig. 1 of Poust et al. [31]) as we observe in specimens IVPP V13352, V13320, STM 5-142, 5–75, 5–5 of *Microraptor* (see Additional file 2: Figs. S2, S5, S3; Figs. 2 and 3). *Changyuraptor* possesses curved tibial feathers projecting with angles of ~ 90°, as observed in Fig. 4 of Han et al. [28]. In *Anchiornis* specimen LPM-B00169, these feathers display an angle of ~ 170° relative to the distal part of the tibiotarsus. This angle is probably less obtuse proximally but the curved tibial feathers are partially obscured by fragmented bones of the pelvic girdle. The Berlin *Archaeopteryx* specimen [50] displays feathers with an angle of ~ 130° along the middle portion of the posterior tibiotarsus.

#### Minor coverts

The shorter tibial feathers form a second layer that we name as minor coverts according to naming conventions for the modern avian forewing. In Zheng et al. [4], these feathers appear to be reconstructed on the tibiotarsus of *Anchiornis* based on LPM B00169 and of *Sapeornis* based on specimen STM 16–18 [56]. Also, they have been reconstructed along the tibiotarsus of the enantiornithine *Cathayornis* based on hind limb feathers in specimen STM 7–50 where patches of feathers are present along the anterior and the posterior hind limb [4]. Along the tibiotarsus of *Microraptor* between 10(STM 5-221) and 15 feathers (STM 5–9) are preserved (see Additional file 2: Figs. S6, S8). However, t there were probably ~ 18 feathers in total based on the spaces where we would expect additional feathers: on the proximal tibiotarsus of STM 5-9and both ends of the tibiotarsus of STM 5-221. These feathers decrease in length distally along the tibiotarsus as seen by a decrease from 68.56 mm to 25.44 mm in specimen STM 6–86 (Fig. 4).

#### Anterior coverts

Along the anterior tibiotarsus, the proximal portion has feathers that appear to be completely plumulaceous, as exemplified by well-preserved feathers in specimen STM 5–9 (see Additional file 2: Fig. S6). Along the mid- and distal portion of the anterior tibiotarsus of specimens STM 5–9 and 6–62 (see Additional file 2: Fig. S6; Fig. 5), the preserved feathers have a plumulaceous base but become pennaceous and symmetrically close-vaned towards the apex. This layer of anterior tibial feathers contrasts with the two layers of asymmetrically vaned tibial feathers previously described in the Berlin *Archaeopteryx* [18]. The Altmühl specimen of *Archaeopteryx* displays tibial feathers that are considerably long (length ranging from ~ 58 mm for the proximal ones to ~ 35 mm for the distal ones) and suggested to be anteriorly implanted [51] with an angle of ~ 90° to the bone. Based on the pennaceous portion of these feathers we were able to measure angles ranging from ~ 120° and ~ 180° to the bone in specimens STM 5-221 and 5–5 respectively (see Additional file 2: Fig. S8; Fig. 3). These feathers increase in lengthproximally. This suggests that they can rotate and twist together, such that the surface created by the feather patch increases to form a continuous airfoil, as proposed for *Archaeopteryx* (see Fig. 10 of Longrich [18]).

### Femoral feathers

#### Long femoral feathers

These feathers are likely symmetrically close-vaned, as seen in *Microraptor* specimen IVPP V13320 in this study (see Additional file 2: Fig. S5) as well as in the controversial specimen LPM-0200 [53]. There are between 7 (STM 5–5) and 8 (STM 6–86) long femoral feathers preserved (Figs. 3 and 4) and we estimate a total of ~ 10 (Fig. 1) based on preservational gaps in the specimens. These feathers vary in size, and they are best preserved in STM 6–86 (Fig. 4) where their length to the bone varies from 95.5 mm on the proximal end to 155.07 mm for the most distal feather preserved, the portion situated at the distal edge of the femur being obscured by the tibial feathers. The angle of these feathers relative to the femur varies between ~ 40° for the proximal and distal ends of the femur in specimen STM 5–5 (Fig. 3) to ~ 70° along the proximal end of the femur in specimen STM 6–86 (Fig. 4). Note that the angle of these feathers does not vary much within a given specimen e.g., in specimen STM 6–86 the angles measure ~ 60° at the distal end and ~ 70° at the proximal end (Fig. 4).

#### Minor coverts

~ 10–15 shorter pennaceous feathers are estimated (Fig. 1) along the femur based on at least 5 missing feathers in specimen IVPP V13320 and STM 5–5 and 6–86 (see Additional file 2: Fig. S5; Figs. 3 and 4). In the same specimens, the length of these feathers varies little, and they appear to follow the contours of the soft tissues. In specimen STM 6–86, where they are well-preserved, they measure between 46.19 mm and 51.06 mm when measured to the bone and slightly decrease in size proximally (Fig. 4). These feathers display a minimum angle of ~ 45° to the proximal end of the femur in STM 5–5 (Fig. 3) to ~ 90° to the proximal and distal ends of the femur in STM 6–86 where the angle varies little (Fig. 4).

#### Anterior coverts

In specimens STM 5-109 and 6–86 (see Additional file 2: Fig. S9; Fig. 4), the anterior side of the femur is covered by feather patches. These are comparable to those of the early-diverging avialan *Yanornis* which are described as plumulaceous by Zheng et al. [4]. The feathers were insufficiently preserved to measure their angle with the femur.

### Non-feather soft tissues

Current knowledge of the non-feather soft tissues of the leg of *Microraptor* is focussed on the foot, although narrow thigh muscles have been proposed based on its short ilium [57], as observed in *Wulong* [31, 58]. Pittman et al. [2] used Laser-Stimulated Fluorescence imaging to reconstruct a bird-like podotheca (*sensu* [59]) for *Microraptor* that features scutate, scutellate and reticulate scales (STM 5-109), as in *Anchiornis* (STM 0-147). The pedal digits of *Microraptor* lack preserved feathers [2], as opposed to the short feathers associated with *Anchiornis* (LPM-B00169) [46]. Here, for the first time, detailed soft tissue descriptions are extended to the rest of the leg.

#### Preservation

Soft tissues are best preserved around the leg bones of specimens STM 5–75, 5–5, 6–86, 5-9and 5-221(see Figs. 2, 3 and 4; Additional file 2: Figs. S6, S8). The patches of soft tissues observed inform the overall shape of the leg, but differences in leg shape between specimens are harder to resolve. The soft tissue outline is thinner around the distal portion of the posterior side of the tibiotarsus compared to its proximal portion, as seen in STM 5–9. These soft tissues are also thinner along the anterior part of the tibiotarsus than along the posterior part, as observed in specimen STM 5–5 (Fig. 3). As preserved in specimen STM 6–86 (Fig. 4), the soft tissues display a uniform width posteriorly with feather rachises implanted within them along the metatarsus. Along the anterior metatarsus of specimen STM 5-221 (see Additional file 2: Fig. S8), soft tissues are obscured by downy feathers and are the widest at its middle portion: decreasing in size proximally and distally. In specimen STM 6–86 (Fig. 4), these anterior soft tissues display a different pattern by being the widest at the proximal portion of the anterior metatarsus and decreasing in size distally. The hind limbs of specimens STM 5-221 and 6–86 are in different positions with the first showing an acute angle between the tibiotarsus and metatarsus and the second showing an obtuse angle between the same bones (see Additional file 2: Fig. S8; Fig. 4).

#### Rachis anchorage

Some rachises are anchored into soft tissues and reach the bone surface, as preserved in STM 5–9 and 5-221 (see Additional file 2: Figs. S6, S8). These observations combined with those made in STM 6–62 (Fig. 5) show that the feathers are closely linked to the bones, so angles between the feathers and bone surface were made accordingly. As preserved in the forelimb, a few feather sheaths are preserved along the hind limb, but they are only visible along the posterior tibiotarsus in specimen STM 5–75 (Fig. 2).

#### Pedal soft tissues

The pedal digital pads of *Microraptor* are arthrally arranged, as preserved in specimens STM 5–75, 5-109 and 5-172 (see Fig. 2 and Additional file 2: Figs. S4, S9). Five other specimens also preserve partial digital pads: STM 5–5, 6–62, 5-142, 5-150, and 5-221 (see Figs. 3 and 5; Additional file 2: Figs. S3, S10, S8). However, there were no soft tissues associated with digit I in our sample and in the literature. Among specimens in this sample, only STM 5–75 partially preserves the claw pad. This covers the claw tubercle of digit II (Fig. 2) and appears to be well-developed In living birds, such as the raptors in Fig. 1 of Fowler et al. [60], the claw pads of all digits are similar, lacking the specialised digit seen in *Microraptor*. In *Microraptor*, three well-developed pads are preserved under digit III [2]. The first pad is separated from the second pad by a broad fold and the second pad is separated from the third pad by a smaller fold [2]. On digit IV of specimen STM 5-109, four pads are observed with the first pad (between phalanges IV-I and IV-2) likely being protrusive and separated by a broad fold from the second pad (see Additional file 2: Fig. S9). On digit IV of STM 5–75, four pads are observed with a furrow separating the first pad from the protrusive tarsal pad (see Pittman et al. [2] and Fig. 2). As described by Pittman et al. [2], there are three different types of scales (STM 5-109): sub-rectangular scutate scales on the dorsal surface of the digits, polygonal scutellate scales situated laterally on the phalanges and dorsolaterally on the digital pads as well as polygonal reticulate scales covering the digital pads. The same reticulate scales on arthrally arranged pedal digit pads have also been observed in *Anchiornis* specimen STM 0-147 [61, 62]. In *Anchiornis* (STM 0-147), scutate scales partially cover the dorsal portion of digit III and the lateral and dorsolateral portions of the digits of the same specimen [2]. In microraptorines, the only other specimen to display pedal pads with reticulate scales is *Sinornithosaurus* specimen NGMC 91 where they cover the second pad of digit II, which shows an arthral arrangement [62].T.

### Skeleton

Here, we provide a detailed anatomical description of the hind limbs of *Microraptor* bone by bone. Better preservation across our expanded sample allows us to clarify the morphology of the femur and tibiotarsus in particular. Body mass calculations display a large disparity in our sample with estimated masses nearly 10 times greater for the heaviest individual than for the lightest one. The pedal claw angle of *Microraptor* displays variations that could potentially be related to different behaviours. By combining observations between the part and counterpart of specimens IVPP V12330 as well as STM 5-109, 5-142, 6–86 and 6–62, all bones of the hind limb are accounted for (see Additional file 1: Table S5; Additional file 2: Figs. S11, S9, S3; Figs. 4 and 5). The total length of the hind limb was measured in 12 specimens ranging from 155.3 mm in specimen IVPP V12330 to more than twice the length in specimen STM 5-142 (327.8 mm). While interlimb ratios (F: T:MT) vary, there is no consistent pattern in relation to total limb size as the smallest specimen IVPP V12330 (32.3: 44.3: 21.1) and largest specimen STM 5-142 (32.8: 45.7: 21.5) have nearly identical ratios (see Additional file 1: Table S6). Thus, we do not see good evidence of interlimb elemental allometry in *Microraptor*.

#### Pelvic girdle

The pelvic girdle of *Microraptor* has been partially described in past studies [17, 25, 27, 31, 58, 63]. Preserved examples include STM 5–9, 5–75, 5-142, 5-150, 5-172, 6–62 & IVPP V13352, and although generally compressed, display derived features similar to those found in other microraptorines like *Sinornithosaurus*, in early troodontids like *Sinovenator* and in early-diverging birds as reported by Xu et al. [17]. These features include a tapered postacetabular process of the ilium (reduced in thickness towards one end), a retroverted pubis, and a short ischium with two dorsal processes and a distally located obturator process.

**Fig. 6:**
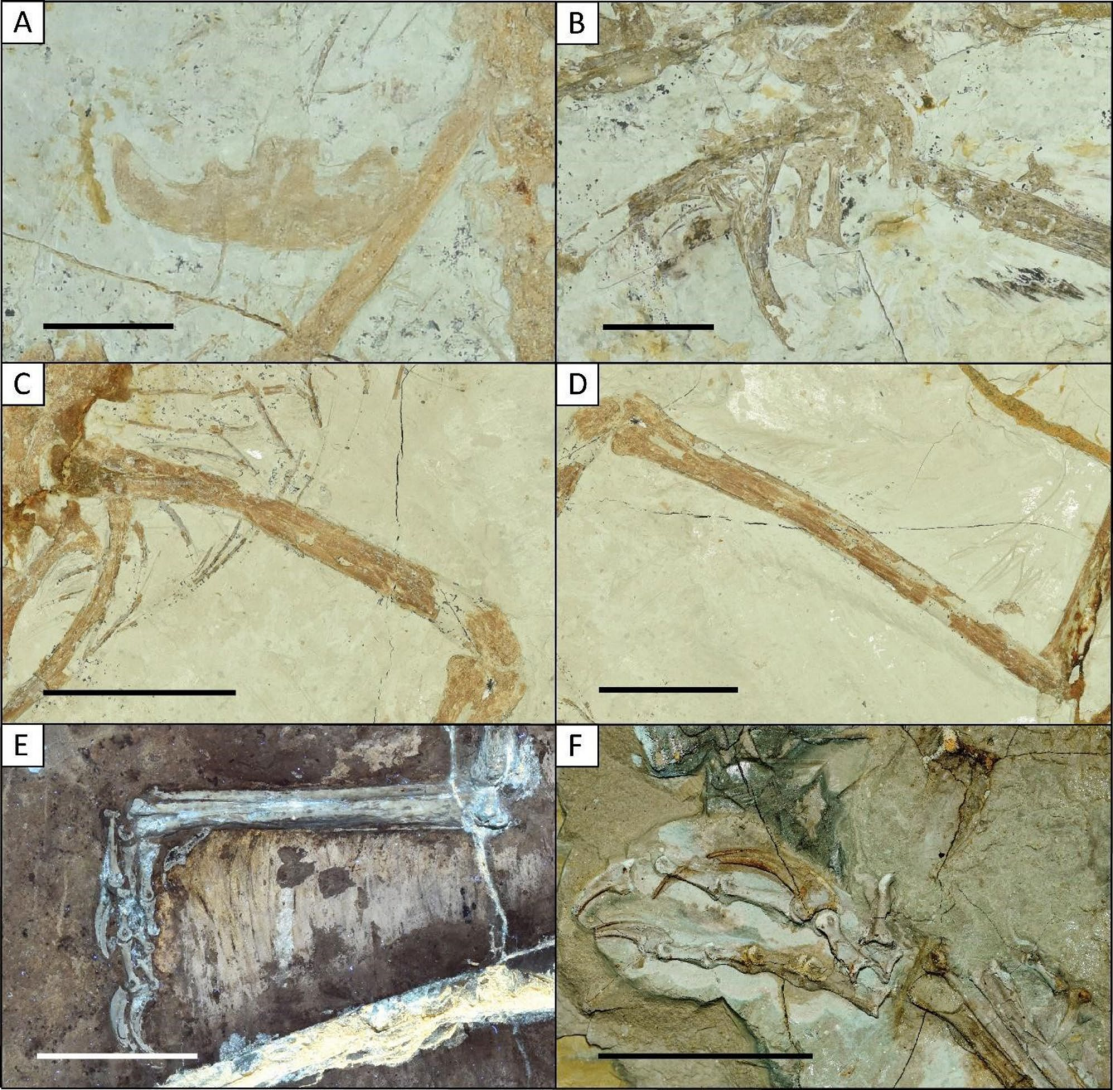
Bone features on the pelvic girdle and hind limbs of *Microraptor*. Well-preserved ilium of 5-172 in lateral view (**A**), pelvic girdle of STM 5–75 with well-preserved ischia and fragmented pubis in lateral view (**B**), well-preserved right femur and right tibiotarsus of STM 5-221 in dorsolateral view (**C**, **D**), well-preserved left metatarsus of STM 6–62 in dorsolateral view with pedal digit linked (**E**), well-preserved left pedal digits of STM 5-109 in lateral view (**F**)

Both ilia are preserved in articulation in lateral view in STM 5–9, 5–75, 5-142 and 5-150 as well as IVPP V13352, with STM 5-150 preserving the articulation in dorsal view. Although both ilia are preserved in STM 5-172, only one is articulated, the other being well-preserved in lateral view (Fig. 6A). A partially articulated ilium is preserved in lateral view in STM 5–75, 5–93 and 6–62. The preacetabular process projects anteriorly in STM 5–9, 5–75, 5-142, 5-150, 5-172 and 6–62, where its anterior portion is rounded and displays an anteroventral hook. The preacetabular process is only slightly longer than the postacetabular process of the ilia which tapers posteriorly as shown in Xu et al. [17], Pei et al. [25], Gong et al. [27] and Poust et al. [31]. This contrasts with early-diverging avialans such as *Archaeopteryx* [64, 65], *Anchiornis* [66] and *Confuciusornis* [67], where the ilium has either a squared or rounded anterior end [26, 68]. The antiliac shelf is short with no distinct cuppedicus fossa present in STM 5–9, 5–75, 5-142 and 5-150. The pubic peduncle is much larger than the ischial peduncle with its ventral margin sloping posteroventrally. The pubic peduncle does not have a cuppedicus fossa like other microraptorines [25, 27, 31, 58, 69] and non-microraptorine dromaeosaurids [68, 70, 71], which differs from early-diverging avialans like *Anchiornis* [66], *Archaeopteryx* [64, 65] and *Confuciusornis* [67]. The acetabulum located at the middle of the ilium is visible in lateral view in STM 5-142 and 5-172 which shows that it is partially closed with no overlying supracetabular crest on the lateral surface, as originally reported by Gong et al. [27] for *Microraptor* specimen LVH 0026.

Paired pubes in STM 5–9, 5–75, 5-142 and 5-172 are fused near the pubic boot. However, the dorsal portions of the pubes are covered by overlapping bones making it difficult to describe this fusion in detail. These paired pubes are preserved in posterolateral view with one pubis overlapping the other as we also observe in the previously published specimens IVPP V13352 [17], BMNHC PH881 [25], and LHV 0026 [27]. Xu et al. [17] described the pubes as retroverted for IVPP V13352, but in our other study specimens we do not have the preservation required to comment on this. We also observe that pubes are much more retroverted in other microraptorines and non-microraptorine dromaeosaurids [26], *Anchiornis* [66], *Archaeopteryx* [64, 65], *Confuciusornis* [67] and *Sinovenator* [72]. The pubic shafts are anteroposteriorly flattened and posteroventrally inclined, resulting in an articulation with the pubic peduncle on the ilium that resembles the opisthopubic condition observed in other dromaeosaurids [26, 31, 58, 73],in the troodontids *Sinovenator* [72] and *Saurornithoides* [74], alvarezsaurids [75–77] and therizinosaurs [78, 79].

The ischia preserved in lateral view in STM 5–9, 5–75 (Fig. 6B), 5-142, 5-172 and 5-221 are proximodistally short and identical to IVPP V13552 [17, 58]. The ischia are flat, unfused, L-shaped in lateral view at both ends, possess a large obturator process with two dorsal processes, and are approximately half the length of the pubes with their shaft curving anteriorly in lateral view, similar to other dromaeosaurids and early-diverging avialans such as *Archaeopteryx*,* Anchiornis* and *Confuciusornis* [31, 58, 65–67]. The pubic processes in all these specimens are much longer than the iliac processes and are separated by a short concave area, as seen in CAGS 20-7-004 and CAGS 20-8-001 [58].

#### Femur

The femur in each specimen is likely bowed laterally and anteriorly, consistent with most dromaeosaurids [26, 27]. In specimen IVPP V13320 (see Additional file 2: Fig. S5) this bowing appears to be exaggerated due to preservation; however, bowing is clearly visible in STM 5-221. The femur is preserved in all studied specimens, except STM 5 − 4. Its length varies: ranging from 51.66 mm in specimen BMNHC PH881 to 108.66 mm in specimen STM 5-142. The trochanters are most clearly visible in specimen STM 5-109 as the preservation is poorer in other study specimens. In *Microraptor*, the greater and lesser trochanters are present and remain separated, similar to *Anchiornis* and *Archaeopteryx* [58, 66]. This trait is also observed in larger fossil paravians, includingthe troodontid *Talos* [80], *Saurornithoides* [74] and *Gobivenator* [81] as well as the dromaeosaurid *Deinonychus* [82]. In contrast, the trochanters are fused in the potential early-diverging microraptorine *Tianyuraptor* and the early avialan *Confuciusornis* as well as in the parvicursorine alvarezsaur *Parvicursor* [83–85].

A femoral accessory crest located at the base of the lesser trochanter has previously been documented in specimens BMNHC PH881, IVPP V12330 and CAGS 20-8-001 [25, 58, 63].Within Microraptorinae, *Zhongjianosaurus* also exhibits this trait [30] as well as the oviraptorosaurians *Caudipteryx*, *Microvenator*, the Caenagnathidae and some oviraptorids [86, 87]. This crest is potentially preserved in *Microraptor* specimens STM 5-221 and 6–86 (see Additional file 2: Fig. S8; Fig. 4), but its presence in other specimens cannot be confirmed as this ideally requires a well-preserved proximal femur visible in dorsolateral view. This anatomical structure is likely associated with the insertion of the *M. pubo-ischio-femoralis internus* 2 group, which is replaced in Aves (Neornithes) by *M. iliotrochantericus cranialis* and *M. iliotrochantericus medius* [87]. In this regard, *Microraptor* more closely resembles non-avian theropods and crocodilians than modern birds [88]. In specimen BMNHC PH881, the lateral and medial condyles of the femur are well-preserved and subequal in size, with the lateral condyle being slightly larger (see Additional file 2: Fig. S1).

Within the study sample, the femoral head is only partially preserved and visible in specimen STM 5-109 (see Additional file 2: Fig. S9), where it is completely detached from the acetabulum. The femoral head has previously been described in *Microraptor* specimen IVPP V12622, where it exhibits a domed shape similar to that of modern birds [89]. However, in all newly examined specimens, the femoral head is either not visible or too poorly preserved to allow reconstruction of its shape (see STM 5-109: Additional file 2: Fig. S9). Hwang et al. [58] previously described the femoral head in specimen CAGS 20-7-004 as possessing a distinct ventral lip, a feature also observed in specimen STM 5-109 (see Additional file 2: Fig. S9). A ventral lip is also preserved in specimen DNHM D2933 of *Wulong* [31] and in the oviraptorosaurian *Elmisaurus* [90]. This contrasts with *Confuciusornis* where the femoral head is round [91], and with *Archaeopteryx* and *Anchiornis*, where it is hemispherical ( [82]; personal observation in Fig. 4 of Xu et al. [92]). The femur of *Microraptor* also features a short neck [23], similar to that of *Anchiornis* (personal observation in Fig. 4. of Xu et al. [92]), in contrast to *Archaeopteryx*, where no distinct neck is present [82] and *Confuciusornis* where it is robust [91]. An elevated femoral head has been proposed as a diagnostic character of Microraptorinae [27]. This feature is observed in *Microraptor* but appears to vary among specimens. In specimen CAGS 20-7-004, the femoral head is slightly higher than the greater trochanter, whereas in specimen STM 5-109, it is significantly higher than the greater trochanter. The greater and lesser trochanters are fused in the potential microraptorine *Tianyuraptor* [85]. Both trochanters are also present in other early paravians, including *Confuciusornis* and *Archaeopteryx* [82, 91].

#### Tibiotarsus

The tibiotarsus is only slightly expanded proximally and is here described as straight (STM 5 − 4: Additional file 2: Fig. S7) to slightly bowed (STM 5-221 and IVPP V13352 Fig. 6D; Additional file 2: Fig. S2), consistent with Pei et al. [25]. However, Turner et al. [26] attributed the presence of a bowed tibia to preservation bias and deformation. Note that a slightly bowed tibia is an autapomorphy for *Microraptor* according to Gong et al. [27]. The tibiotarsus is preserved in all studied specimens, with a length ranging from 69.68 mm in specimen IVPP V12330 to 149.96 mm in specimen STM 5-142.

The tibiotarsus: femur ratio was calculated for at least one leg among the 12 study specimens where both bones are well-preserved along their entire length (see Additional file 1: Table S6). This ratio ranges from 1.14 in STM 6–62 to 1.48 in specimen STM 6–86. When correlated with specimen size, the ratio increases with specimen size. Specimens CAGS 20-7-004 and 20-8-001, studied by Hwang et al. [58], exhibit ratios of 1.26 and 1.28 respectively, with estimated total body lengths of 387.51 mm (with sacral vertebrae missing) and 315.75 mm (without the head and a few missing dorsal vertebrae). These ratios are close to those calculated with our study sample. Comparison with the holotype specimen of *M. hanqingi*, LVH 0026, which is 810.35 mm long based on new measurements, reveals a tibiotarsus: femur ratio of 1.41. This ratio is comparable to that of the longest individuals in this study, such as STM 5-142 and 6–86 (see Additional file 2: Fig. S3; Fig. 4) [27]. According to Christiansen and Bonde [93], birds exhibit proportionally longer tibiotarsi and metatarsi relative to femora distinguishing them from non-avialan theropods. This trend is also observed in *Anchiornis* specimens IVPP V14378 and LPM-B00169, which have tibia: femur ratio of 1.57 and 1.6 respectively, higher than those calculated for *Microraptor* [46, 92]. Gatesy [94] calculated a range of theropod femur: tibia ratios and suggested that birds exhibit a decrease in this ratio with increasing limb length, whereas this ratio tends to increase in non-avialan theropods with their limb length. Our data do not show a clear pattern in this regard, as the smallest specimen in our sample, BMNHC PH881, exhibits a slightly lower (1.38) than LVH 0026 and STM 6–86. This discrepancy may be due to our sample capturing a more limited range of body sizes, likely skewed towards subadults and adults.

Specimen D-2842 has a femur length of 86 mm and tibia length of 118 mm that yields a tibia/femur ratio of 1.37. This ratio was compared to other paravians (see Additional file 1: Table S7): it is similar to the 1.36 ratio of dromaeosaurid *Wulong bohaiensis* from the Early Cretaceous of China (D-2933; [31]), the ratio of 1.34 of *Serikornis sungeri* (subadult) from the Late Jurassic of China (PMOL.AB00200; [95]; yields hind limb osteology data) as well as the 1.42 ratio of *Aurornis xui* (adult; considered a junior synonym of *Anchiornis huxleyi* by Pei et al. [66]) from the Late Jurassic of China (YFGP-T5198; [95]). Specimen D-2842 has a similar femur length to *Jeholornis* sp. (adult; STM 2–51; 88 mm; [96]) from the Early Cretaceous of China. Smaller taxa providing data on hind limb osteohistology include early paravians such as *Serikornis sungeri* (subadult) from the Late Jurassic of China (PMOL.AB00200; [95]) with a ratio of 1.34, *Anchiornis huxleyi* (subadult) from the Late Jurassic of China (YFGP-T5199; [95]) has a comparatively higher ratio of 1.55, similar to *Eosinopteryx brevipenna* (juvenile) from the Late Jurassic of China (YFGP-T5197; [95]) which has a ratio of 1.6. Both of these small taxa have been sampled for hind limb osteohistology. Larger taxa that have been sampled in this way include *Sinornithosaurus* ´*haoiana*´ from the Early Cretaceous of China (late juvenile; D-214; [31]) with a tibia measuring 143 mm, *Changyuraptor yangi* from the Early Cretaceous of China (adult; HG-B016; [28]) with a femur measuring 153 mm,*Buitreraptor* from the Late Cretaceous of Argentina: (subadult; MPCA-PV-598; [97]) with a tibia/femur ratio of 1.33, *Dakotaraptor* and from the Late Cretaceous of USA (adults; [98]) with PBMNH.P.10.113.T having a tibia/femur ratio of 1.2 and PBMNH.P.10.115.T having a femur measuring 485 mm.

#### Fibula

The fibula is likely proximally expanded and rapidly thins to a splint distally to adhere to the tibiotarsus. The fibula is at least partially preserved in specimen BMNHC PH881 as well as in STM 5–9, 5-150, 5–75, 6–86 and 6–62 (see Additional file 2: Figs. S1, S6, S10; Figs. 2, 4 and 5).

#### Metatarsus

The metatarsus is measurable in at least 13 specimens, with its length estimated in STM 5 − 4, where the distal half is missing, and in STM 5–5, where the metatarsi are highly fragmented (see Additional file 2: Fig. S7; Fig. 3). The metatarsus length ranges from 33.77 mm in specimen IVPP V12330 to 70.5 mm in specimen STM 5-142. The subarctometatarsalian condition [17] is visible in nearly all specimens, except for STM 5–5 (Fig. 3) where the metatarsals are broken and metatarsal III is difficult to observe [99]. However, this feature is well-preserved in specimens STM 6–62, 5-109 and 5-221 (see Fig. 6E; Additional file 2: Figs. S9, S8). Specimens IVPP V13320 and V13352 as well as STM 5–9 and 5-221 exhibit metatarsals II and IV of similar length (see Additional file 2: Figs. S5, S2, S6, S8). This characteristic was proposed as a diagnostic feature of *Microraptor hanqingi* by Gong et al. [27], though it is also present in the holotype of *M. gui* [17]. In the holotype specimen IVPP V12330 of *M. zhaoianus* [26] metatarsals III and IV are similar in length, while metatarsal II is shorter; however, preservation is poor (see Additional file 2: Fig. S11). A similar condition is observed in specimen STM 5-109 (see Additional file 2: Fig. S9). In specimen STM 5-172, metatarsals II, III and IV are equal in length (see Additional file 2: Fig. S4). In STM 6–62, metatarsal II is slightly shorter than metatarsal IV on one side (Fig. 5). At least one complete or nearly complete metatarsal V is preserved in specimens BMNHC PH881, IVPP V13352 and STM 5-150 (see Additional file 2: Figs. S1, S2, S10). Its length is over 50% of the length of metatarsal IV, a trait proposed as diagnostic for Microraptorinae [23]. Metatarsal IV is straight but slightly shorter and more robust than metatarsal III, with a squared distal end in specimens BMNHC PH881 and STM 5–9. A similar condition is likely present in other specimens with a poorly preserved metatarsal IV (see Additional file 2: Figs. S1, S6). Metatarsal V exhibits an elongated and bowed structure in BMNHC PH881 and in STM 5–9 and 5–75 (see Additional file 2: Figs. S1, S6; Fig. 2). This elongation has been mentioned as a feature that refers *Microraptor* to the Dromaeosauridae [17]. Metatarsal V is also proximally straight before bowing distally [58] in specimens BMNHC PH881, IVPP V13352 and STM 5-150. Metatarsal I is preserved in specimens IVPP V12330, V13320 and V13352 as well as specimens STM 5–9, 5–93, 5-142, 6–62 and 6–86 (see Additional file 2: Figs. S11, S5, S2, S6, S12, S3; Figs. 4 and 5).Its position, with the ungual of the pedal digit I reaching the midpoint of the pedal phalanx II-1, is consistently more distally positioned than in other dromaeosaurids such as *Velociraptor* [100].

The metatarsus: femur was calculated, yielding values ranging from 0.6 in STM 6–62 to 0.77 in IVPP V13320. While there is a slight tendency for smaller taxa to show larger ratios than larger taxa, this is not statistically significant (Mt/F: F, slope − 0.001, r2 = 0.14, p (uncor) = 0.17). Data collected by Benson and Choiniere [101] indicate that *Deinonychus* (Dromaeosauridae) has a lower ratio of 0.51 comparable to the avialans *Patagopteryx* (0.51) and *Sapeornis* (0.56), as well as several other cursorial theropods including *Coelurus*, *Velociraptor*, *Buitreraptor*, *Ingenia* amongst others [102]. Conversely, *Microraptor* has a ratio lower than *Caudipteryx* (Oviraptorosauria) which has a value of 0.78 according to the data from Benson and Choiniere [101].

#### Phalanges

According to previous observations, the phalangeal count is 2 for digit I, 3 for digit II, 4 for digit III and 5 for digit IV [63]. Strongly ginglymoid interphalangeal joints have been previously described in digits II, III and IV of specimens STM 5-109 and 5-172 [2]. In the same study, other elements such as sagittal furrows and hinge-like distal articulation facets are also described for STM 5-109 and 5-172. The digits are best preserved in specimens IVPP V12230 and STM 5-109, 5–75, 6–86 and 6–62 (see Additional file 2: Figs. S11, S9; Figs. 4, 5 and 6F). Some specimens display a digit II with only 2 phalanges connected: IVPP V12330 and V13320 as well as STM 5-150, 5-172, 6–86 and 6–62 (see Additional file 2: Figs. S11, S5, S10, S4; Figs. 4 and 5) which is most likely due to preservation bias as well as for specimens STM 5–93, 5-142 and 5-221 where it is clearly due to a preservation bias (see Additional file 2: Figs. S12, S3, S8). It is tough to see the number of phalanges in digit II of specimen STM 5–9 (see Additional file 2: Fig. S9) and specimen IVPP V13352 displays 2 phalanges on the right digit II and 3 phalanges on the left one (see Additional file 2: Fig. S2). Also, specimen CAGS 20-8-001 has been described with 3 phalanges in Hwang et al. [58] and the same characteristic is observed in other microraptorines such as *Changyuraptor* and *Wulong* [28, 31, 82, 92] as well as certain other early paravians including *Anchiornis* and *Archaeopteryx*.

The length of the digits differs between specimens, but in most specimens, digit III is the longest followed by digit IV, II, and I respectively, as observed in *Anchiornis* [92]. This differs in specimen STM 6–86 where digits III and IV are nearly equal (Fig. 4). This last feature has also been observed in the microraptorine *Wulong* [31]. Digit II is specialised in deinonychosaurians with an enlarged pedal ungual [23] and is only missing in specimens BMNHC PH881 and STM 5 − 4 (see Additional file 2: Figs. S1, S7). This is different in other early avialans like *Archaeopteryx* and *Confuciusornis* where this differentiation is absent [82, 91]. Pedal phalanges III-1 and IV-1 are likely much more robust than distal phalanges in most *Microraptor* specimens such as IVPP 13,320 and LVH 0026, except STM 5 − 4, 5-142, 5-221 and 5–5 where they are poorly preserved (see Additional file 2: Figs. S5, S7, S8; Fig. 3). So, this character is potentially not only diagnostic of *M. zhaoianus* (*contra* [25]) underscoring the need for further taxonomic clarification of *Microraptor*. Digit I is at least partially preserved in 8 specimens studied and is missing in IVPP V13320 and V13352 as well as STM 5 − 4, 5–93, 5-150, 5-1725-221 and 5–75 (see Additional file 2: Figs. S5, S2, S7, S12, S10, S4, S8; Fig. 2). The proximal positioning of the digit I in IVPP V12330 and STM 6–62 (see Additional file 2: Fig. S11; Fig. 5) is consistent with the fact that the digit I could not work in opposition to digit II to grip prey [2].

Pedal unguals are strongly recurved and slender and they display a prominent flexor tubercle. This trait has been described in most microraptorines but is missing in *Zhenyuanlong* [33] and in *Tianyuraptor* according to new observations on specimen STM 1–3. Keratinous claw sheaths are at least partially preserved in 14 specimens (see Additional file 1: Tables S5, S8) and are fully missing in specimens STM 5 − 4 and 5–9, the latter having no preserved digits. The pedal unguals of digits III and IV are quite similar and they are missing in specimens IVPP V13320 and V13352 and in STM 5 − 4. The outer angle with the claw sheath has been measured between ~ 150° and ~ 160° on pedal unguals II, III and IV in specimen STM 5–75 and between ~ 110 and ~ 130° on the same pedal unguals in specimen IVPP V12330, according to the methodology of Pike and Maitland [39]. On the same specimens, measurement on same pedal unguals without the claw sheaths are respectively between 100 and ~ 115° for specimen STM 5–75 and between ~ 50 and ~ 70° for specimen IVPP V12330. The only possible measurement of the pedal ungual of digit I was made on specimen IVPP V12230, where the angle is different from the other digits and measures ~ 80° with the claw sheath and ~ 30° without it.

#### Hind limb and trunk length comparisons

The hind limb: trunk ratio has been calculated in 9 specimens where the trunk is measurable and at least one hind limb is preserved along its length. The trunk is measured from the first dorsal to the anterior rim of the acetabulum [93]. This ratio ranges between 2.04 in specimens BMNHC PH881, STM 5–9 and 5-150 to 2.44 in specimen STM 5-172. Specimens BMNHC PH881, IVPP V13320, and STM 5–9, 5-142, 5-150, 5-221 and 6–86 display a ratio between 2 and 2.3, which is similar to *Caudipteryx* (2-2.13). Specimens STM 5–75 and 5-172 display a ratio above 2.3. These ratios are far above those of other non-avialan dinosaurs (0.79–1.55) within the range of modern birds (1.78–2.95) [93].

#### Body mass

The lightest individual was found to be IVPP V12330 with a mass of 0.213 kg and the heaviest was found to be specimen STM 5-142 with a mass of 2.13 kg. According to the results of Benson et al. [41], femoral length is likely not the most accurate method to estimate the minimum circumference around the femoral shaft and thus to calculate body mass. To more accurately estimate it, we use femoral mediolateral shaft diameter. By correlating body mass and the metatarsal remex: femoral length ratio, we observe that the second lightest specimen BMNHC PH881 has the longest metatarsal remiges compared to its femoral length, with a metatarsalremex: femoral length ratio of 2.32.On the opposite end of the spectrum, one of the heaviest specimens STM 6–62 (Fig. 5) has the shortest metatarsal remex compare to its femoral length, with a metatarsal remex: femoral length ratio of 1.32.

#### Osteohistology

**Fig. 7:**
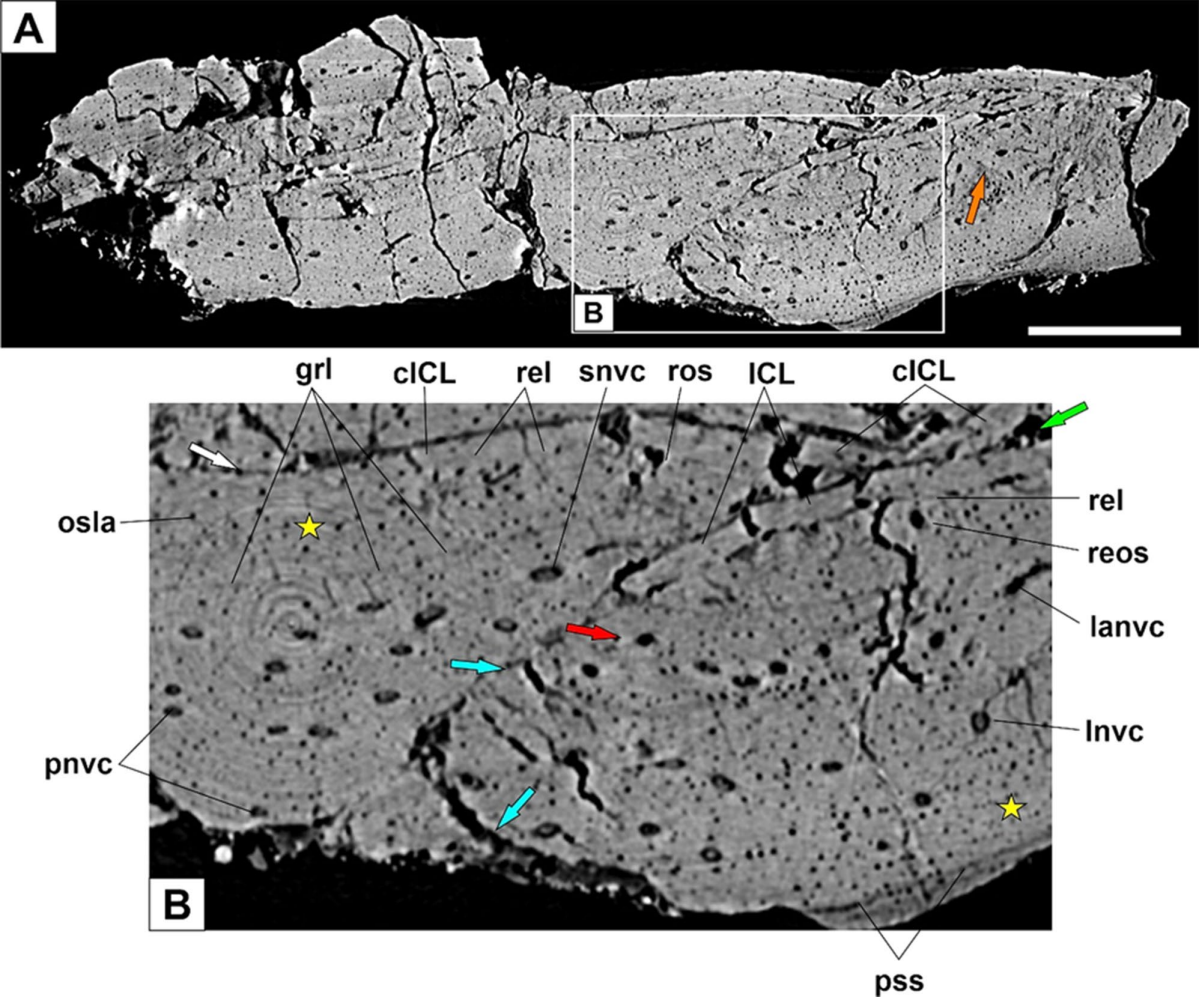
Virtual stylopodium histology of the early-diverging dromaeosaurid *Microraptor*. Synchrotron microtomography of the mid-diaphysis cortex sample of the D-2842 femur; note the presence of secondary osteon (orange arrow) (**A**). Close-up of the cortical bone (**B**); note avascular periosteal regions (asterisk) and a circumferential ring of the primary osteons (red arrow). Endosteal bone of the opposite sides lined up (white arrow) due to the bone collapse leaving a slit-like medullary space in between (green arrow). The collapse led to the cortical fracture (blue arrow). Abbreviations: cICL, counterpart inner circumferential line; grm, growth mark; ICL, inner circumferential line; lanvc, laminar neurovascular canal; lnvc, longitudinal neurovascular canal; osla, osteocyte lacuna; pnvc, primary neurovascular canal; pss, periosteal surface; rel, resorption line; reos, resorption line; snvc, secondary neurovascular canal. The scale bar is 500 microns

**Fig. 8:**
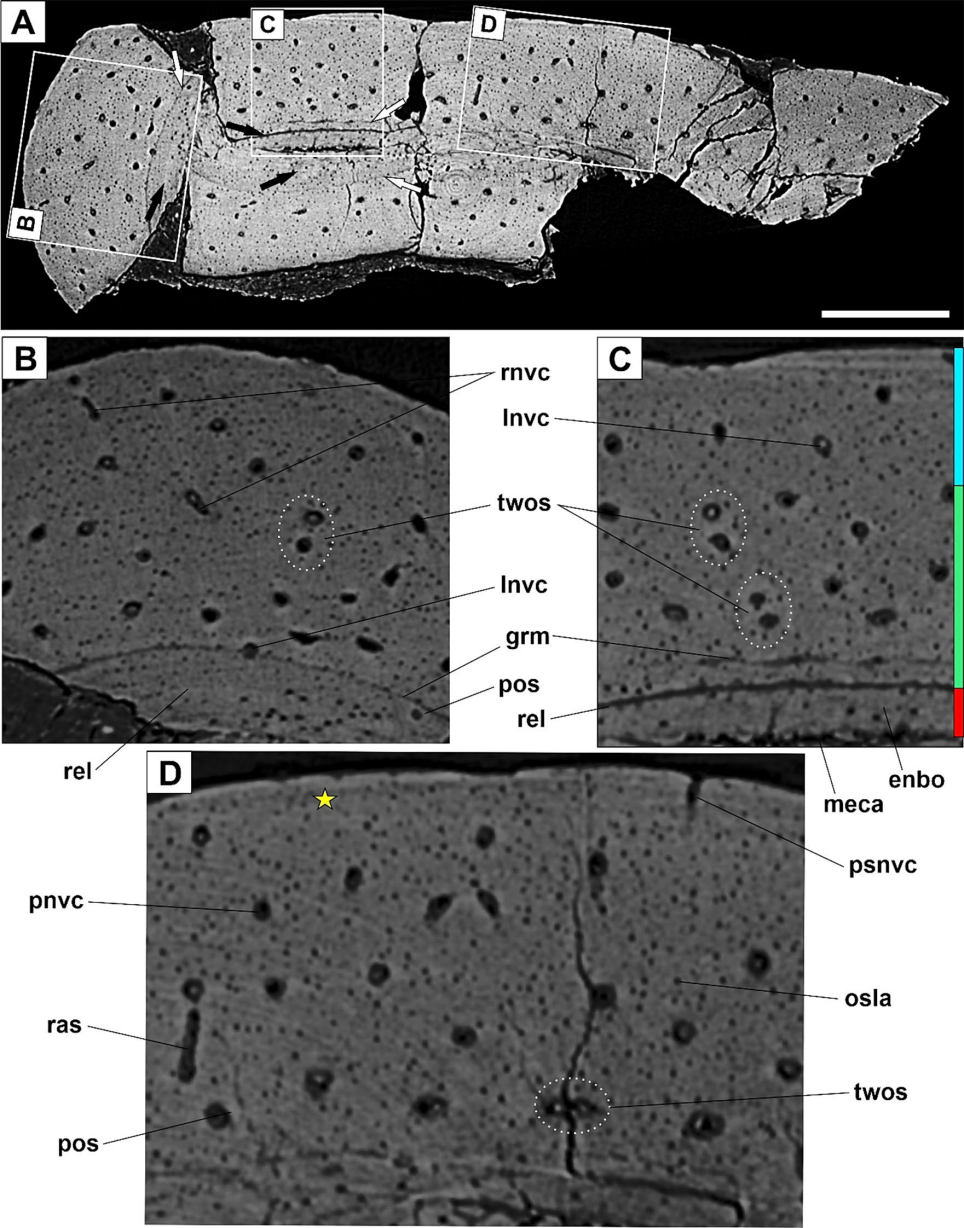
Virtual zeugopodium histology of the early-diverging dromaeosaurid *Microraptor*. Synchrotron microtomography of the mid-diaphysis cortex sample of the D-2842 tibia; note the growth mark (white arrow) versus resorption front (black arrow) (**A**). The close-up of the bent cortical bone shows partly resorbed primary osteon and thick endosteal bone (**B**). The close-up of flattened cortical bone shows three major divisions of the cortex including the endosteal bone (ICL, red column), the mid-periosteal cortex with higher vascularity (green column) and paired osteons (dashed line), and the periosteal cortex with reduced vascularity (blue column) (**C**). Close-up of the neighbouring cortex that exhibits an increased periosteal avascularity together with the neurovascular canal open on the bone surface (**D**). Abbreviations: enbo, endosteal bone; grm, rowth mark; ICL, inner circumferential line; lnvc, longitudinal neurovascular canal; meca, medullary cavity; osla, osteocyte lacuna; pnvc, primary neurovascular canals; ponvc, periosteally open neurovascular canal; pos, primary osteon; ras, radially protecting vascular anastomosis; rnvc, radial neurovascular canal; rel, resorption line; reos, partially resorbed osteon; twos, pair of two closely associated osteons. The scale bar is 500 microns

Here, we examined for the first time the hind limb bone histology of *Microraptor* using virtual transversal sections through the mid-diaphyseal part of the femur and tibia. The mid-diaphyses collapsed post-mortem due to taphonomic reasons. The femoral remains are considerably weathered and crushed (see Fig. 7A and blue arrows in Fig. 7B) with a single profile of the bone cortex available to study (Fig. 7B). Preservational conditions, however, are more intact (Fig. 8A) in the tibia and enable investigation of histological variability within the sampled region (Fig. 8B-D).

### Femur

The studied periosteal region of the femoral cortex is approximately half a millimetre thick (515 μm thick). The innermost periosteal region (known as the perimedullary region) is lined by a thin layer of avascular lamellar bone. This layer constitutes the endosteal region of the femoral cortex (labelled here as the inner circumferential layer; see ICL or cICL in Fig. 7B) forms about 7% (= 40 μm) of the total cortex but varies in thickness from 32 μm to 45 μm. There are no traces of trabecular structures between the collapsed inner circumferential layers (see the white arrow in Fig. 7B) suggesting the presence of a well-differentiated medullary cavity (see the green arrow in Fig. 7B).

The majority of the periosteal cortex comprises fibro-lamellar tissue with randomly distributed osteons. Neurovascular canals are projected longitudinally; a laminar direction is found sporadically in the inner periosteal cortex. A diffuse growth mark (see grl in Fig. 7B) divides the periosteal cortex to the inner (partly resorbed) zone (a third of the cortical bone) and the outer (unfinished) zone. Given its diffuse nature and punctuation by osteocyte lacunae, we found it reasonable to refer to this growth mark as an annulus rather than a line of arrested growth.

It is noteworthy to mention an unusual pattern in the distribution of neurovascular canals in the cortex of *Microraptor* D-2842. Three domains of the periosteal cortex can be recognised. First, large avascular areas (see the asterisk in Fig. 7B) occur between a few primary or secondary osteons in the inner cortex before annulus formation. Second, the middle domain with the highest density of neurovascular canals, either randomly distributed or concentrated into a layer that follows the formation of the annulus (see the red arrow in Fig. 7B). Third, the outer domain with a significant decrease in vascularity (presence of ill-developed osteonal bone) and a more regular distribution of osteocyte lacunae, arranged into slightly arching lines. There is no external fundamental system (EFS).

A few secondary osteons, based on measurements (for example neurovascular canal 42 μm x 23 μm in size *versus* 22 × 18, 23 × 21, 29 × 21, and 30 μm x 25 μm in primary osteons) and osteonal texture (lamellar bone with a maximum diameter of 142 μm), occur in the inner part of the periosteal cortex (see the orange arrow in Fig. 7A).

### Tibia

The cortical bone of the tibia shows a general pattern similar to that of the femur of *Microraptor* D-2842. There are, however, some differences regarding the characteristics of the periosteal bone. Most of the cortical profile is preserved, except for a small portion. The endosteal bone is slightly detached from the periosteal cortex in some places whereas it firmly adheres to the cortex in other positions making its recognition more difficult (check the black arrows in Fig. 8A). The endosteal bone is thicker (58 μm to 64 μm) than that of the femur, forming 10 to 12% of the total cortex. The medullary cavity is mostly obscured due to taphonomic flattening, and it was likely free of cancellous bone.

The thickness of the periosteal cortex varies from 453 μm to 556 μm; it is interrupted by a growth mark preserved in a similar position as that in the femur. The growth mark has a composite structure including characteristics of a line of arrested growth and an annulus, depending on circumferential position. Moreover, it may also be interrupted (check the white arrows in Fig. 8A). The periosteal cortex comprises fibro-lamellar tissue with sparsely and randomly distributed individual primary osteons (with a diameter ranging from 21 μm x 22 μm to 19 μm x 27 μm) and occasional pairs of osteons localised in the inner through the middle (most vascularised) part of the cortex (Fig. 8B-D).

As in the femur, three domains of the periosteal cortex can be recognised in the tibia, although small differences exist. Longitudinal canals are a dominant component of the bone vasculature system, although relatively frequent radially projected canals and sporadic vascular canals open on the bone surface are present (Fig. 8D). The diaphyseal cortex shows a minor drop in vascularity towards its outer surface. This is not coincident with any significant change in the random distribution or density of osteocyte lacunae across the preserved periosteal cortex. There is no external fundamental system developed yet in the femur of the *Microraptor* D-2842. We also did not observe any bone remodelling in the specimen such as intracortical osteoclastic activity and secondary osteons.

## Discussion

### Feather features and locomotory role of Hind limbs

#### Implications of feather organisation

*Microraptor* exhibits a well-developed pennaceous feathering pattern characterised by a hierarchical arrangement along the hind limb, which contrasts with the feather organisation of the forewing. The primary distinction lies in the number of feather layers present on each limb: a maximum of three posterior layers along the metatarsus, whereas the forelimb possesses up to five layers, as detailed by Grosmougin et al. (in this Collection). Another key difference is that certain feathers of the forelimb are directly associated with the digits, a feature absent in the hind limb.

The hind limb feathering pattern of *Microraptor* is unique in both size and structural organisation with no equivalent in the fossil record or among modern birds. However, comparisons can be drawn between the feather arrangement of the hind limb in *Microraptor*, such as the number of feathers and layers, and that of the early paravian *Anchiornis*, for which multiple specimens with preserved hind limb feathers have been discovered and partially described [46, 66, 92]. The hindwing feathering arrangement consists of three layers of posteriorly-directed feathers along the posterior metatarsus, two layers of posteriorly-directed feathers along the posterior tibiotarsus, two layers along the posterior femur and at least 1 layer along the anterior tibiotarsus with plumulaceous feathers covering the remaining anterior leg. Certain feather types exhibit structural similarities to those of the forewing such as the asymmetrically vaned metatarsal remiges and the long metatarsal coverts. The anterior feathers form a continuous sheet that streamlines the hind limb when held in a parasagittal posture, as proposed by Sullivan et al. [6] for the controversial specimen LPM-0200. Furthermore, the long metatarsal coverts and tibial feathers are arranged to outline the shape of the hind limb when abducted.

Regarding hindwing positioning, the findings of this study do not contradict the plausibility of vertical and V-shaped hind limb positions during flight, forward-projecting hind limb positions during hunting and landing, or the absence of extreme abduction ( [89, 103]; *contra* [17, 104]).

If we look at modern birds, we observe that some species display pennaceous feathers along their whole tarsometatarsus. We found this feature in some species of Galliformes (e.g. *Dendragapus obscurus*, *Lagopus lagopus*), Accipitriformes (e.g. *Aquila heliacal*, *Buteo lagopus*), Strigiformes (e.g. *Bubo bubo*, *Strix nebulosa*) and Trogoniiformes (*Pharomachros mocino*). Plumulaceous or pennaceous feathers are present on a portion of the tarsometatarsus of Columbiformes (e.g. *Patagioenas cayennensis*), Caprimulgiformes (e.g. *Chordeiles minor*, *Nyctiphrynus ocellatus*), Bucerotiformes (e.g. *Anthracoceros coronatus*), Piciformes (e.g. *Campephilus principalis*, *Dryocopus pileatus*), Falconiformes (e.g. *Falco sparverius*, *Herpetotheres cachinnans*) and Passeriformes (e.g. *Paradisaea minor*). All the modern birds in our sample have feathers on their tibia and femur, although the femur feathers can often be obscured by the body feathers. But if we compare these modern species with *Microraptor*, their pennaceous feathers are less developed over the hind limb, especially along the tarsometatarsus. Further study of live birds, bird skins as well as dissections would be invaluable in deepening future comparisons. The hindwing of *Microraptor* fundamentally contrasts with well-known examples in other early paravians such as *Anchiornis*,* Sapeornis*,* Archaeopteryx* and *Pedopenna* [4, 46, 48] where the leg feathers are much shorter. *Anchiornis* and *Pedopenna* had shorter feathers without any asymmetrical structure (46, 48). *Sapeornis* and *Archaeopteryx* display leg feathers that are even shorter and may have been less important for weight support and/or control during their flight than for the previous taxa. These differences among early paravians were used to suggest that there was a gradual loss of the distal feathers in the hind limbs of coelurosaurian dinosaurs [4], but this needs to be strictly tested in a phylogenetic context using a broader sample size. The difference in size observed between the metatarsal remiges of BMNHC PH881 could be correlated to sequential moulting [55], but a closer look indicates that it is likely due to preservation bias. Here, the tip of a feather has likely been cleared during the fossilisation process. No specimen of *Microraptor* or any long feathered paravian displays sequential moulting pattern as proposed in the forelimb of *Microraptor* specimen IVPP V13352 [55]. If we look at other pennaraptoran specimens that preserve leg feathers, we find that *Sinornithosaurus* (Dromaeosauridae), which is slightly bigger than *Microraptor*, possesses some short feathers along its proximal hind limbs [105]. We also find badly preserved feathers associated with the tibia of *Yi qi* (Scansoriopterygidae) [106]. In the oviraptorosaurs *Caudipteryx* and *Similicaudipteryx*, we did not observe any leg feathers, but preservation bias cannot be ruled out at present [107]. This suggests that hind limb feathers diversified and evolved multiple times within the paravian clade.

#### Aerodynamic significance of feathers

Vane asymmetry in the primary feathers, especially distal primary feathers, has been found to correlate with volancy in modern birds [108]. As such, the long asymmetrical feathers in *Microraptor* are consistent with an ability to fly, including our observation of long metatarsal covert asymmetry for the first time. That said, the specific aeromechanical implications (if any) of the asymmetric vanes in *Microraptor* are unknown at this time. To date, the only known aeromechanical effects of vane asymmetry in modern birds relate to aeroelastic stability and stall resistance in distal primaries – effects that only occur at vane asymmetries above 3:1 [19]. Since *Microraptor* hindwings lacked distal remiges and lacked metatarsal feathers with vane asymmetries above 3:1, the specific functional implications of vane asymmetry in the hindwing remain a mystery at this time. Furthermore, we confirm that the metatarsal remiges of *Microraptor* have small trailing vane barb angles as observed in *Archaeopteryx* as well as small cutting-edge leading vane barbs as seen in some early paravians, including *Archaeopteryx*, *Sapeornis* and *Confuciusornis* [19].

According to O’Connor and Chang [56], the curved shape of the tibial feathers is linked to a wide separation of the tip of the feathers that they considered to be inconsistent with an aerodynamic function. However, this argument alone is insufficient to exclude a role in flight control since, while a wing without a coherent tip is inefficient, it can still produce meaningful fluid forces.

#### Evidence of feather folding suggests new ecological inference

Many hind limb feather types including the metatarsal remiges, long metatarsal coverts and short coverts display a variation in their projection angle relative to the metatarsus. In modern birds, the erection of feathers is controlled by 2 groups of muscles: the smooth erector feather muscle group and the smooth depressor feather muscle group [109]. If also present in *Microraptor*, this would suggest that *Microraptor* could fold its feathers dorsally to enable greater cursorial locomotion, as we suggest for IVPP V13352. This would also open up its lifestyle beyond the arboreal one that has been traditionally favoured (e.g. Xu et al. [17]) with better cursorial capabilities. This feather configuration is probably related to how the rachises are erected and depressed by muscle contraction along the metatarsus, and other arguments such as feather tract arrangement and direct muscle preservation could potentially be unveiled by LSF technology in the future (*sensu* [24, 61]).

#### Relation between feather growth and flight capabilities

In modern birds, such as Tree Swallows, primaries increase in size even after the stage of development when maximum body mass is reached with seemingly no relationship between feather growth and body mass [110]. Interestingly, one of the biggest specimens in our sample, STM 6–62 (Fig. 5), has a metatarsal remex: femur ratio of only 1.32 and an estimated mass of ~ 1.5 kg. This makes it notably heavy for the size of the feathers and feather surface. This is consistent with the data of Dececchi et al. [111] where it is mentioned that *Microraptor* (as well as other early paravians) had a heavier body than modern birds with a similar wingspan and consequently had higher minimum flight speeds (and likely relatively higher cost of transport). But a substantially bigger sample is required to further test this statement because big specimens such as STM 5–5 (Fig. 3) and STM 6–86 (Fig. 4) have longer metatarsal remiges and a metatarsal remex: femoral length ratio similar to small specimens such as STM 5–75 (Fig. 2).

#### Review and statement on ornamental role of Hind limb feathers

In *Anchiornis*, an ornamental function of the hind limb feathers has been described by Li et al. [112] based on the feathering colour pattern of specimen BMNHC PH828. In *Microraptor*, the hind limb feathering has been suggested to be predominantly iridescent based on BMNHC PH881 and an ornamental function proposed [16]. However, Sullivan et al. [6] stated that predominantly iridescent feathering does not allow for an exclusive ornamental role of the hind limbs by implying that feathering patterns with various colours have a stronger ornamental role (e.g. *Anchiornis* specimen BMNHC PH828; [112]). However, by looking at modern birds, we see that iridescent feathering can be consistent with an ornamental role such as in social birds like the African starlings (Sturnidae) and in non-social birds such as hummingbirds [113, 114]. These arguments combined with the structure of the hindwing of *Microraptor* are not sufficient to demonstrate an exclusive ornamental role of the hindwing, but they do not dispute this possibility in addition to its strong aerodynamic role.

### Soft tissue organisation and ecological inference

#### General shape depicted

Soft tissues found around the femur and tibiotarsus likely relate to the M. iliofibularis/M. quadriceps and M. gastrocnemius, respectively [61]. Based on the portions of their outline that are preserved around the femur of STM 5–5 and around the tibiotarsus of STM 5-221 (see Additional file 2: Fig. S8; Fig. 3), *Microraptor* has the same drumstick shaped leg as *Anchiornis* [61] with important muscle mass around the femur and muscles of the tibiotarsus thinning along the distal end.

#### Implications on hunting style

The soft tissue outline under the metatarsals of STM 6–86 (Fig. 4) would have constrained muscles of the digital flexor group [115] that connected to the plantar pads. Strongly ginglymoid interphalangeal joints provided *Microraptor* with good grasping capability and protrusive pads helped the claws penetrate their prey by acting as supplementary ‘fingers’ [2]. This is emphasised by the fact that *Microraptor* was previously recovered as having a similar hunting strategy on flying prey as modern restraining raptors [2]. By looking at other dromaeosaurids such as *Deinonychus*, we see that their enlarged digit II likely had a pinning role [2, 116]. If we combine the characteristics of modern raptors and other dromaeosaurids, we can assess that *Microraptor* was not able to grasp its prey because of its weak digit I but was probably able to strike prey with its digit II (perhaps while flying). The pad of digit II is well-developed as already observed in specimen STM 5–75 (Fig. 2) and could have a combined role with the metatarsus in resting its body weight prey [2, 116].

As previously described by Pittman et al. [2], *Microraptor* and *Anchiornis* have quite different feet with different ecological implications for their lifestyle and hunting strategy. *Anchiornis* lacks protrusive pads and displays weakly ginglymoid interphalangeal joints which suggest a more ground-dwelling lifestyle, whereas *Microraptor* seems to have hunted from the air. Thus, as in modern ornithophagous raptors, *Microraptor* is expected to have preyed on a variety of different animals as evidence by a range of preserved meals: birds, fishes, lizards and gliding mammals [117].

### Skeleton features implying a complex locomotor role

#### Bone length and implications for flight

In our study we observed that the longest hind limbs seem to be in the longest specimens and thus the tibiotarsus: femur ratio likely increases as limb length increases. This means that *Microraptor* is potentially more convergent with birds than with non-avialan theropods concerning this trait [94]. By comparing hind limb and forelimb lengths in *Microraptor* with those of other early paravians such as *Archaeopteryx* and *Confuciusornis*, we find a pattern consistent with the forelimb elongation seen in Avialae [118]. Information provided by the feathers coupled with observations of the incomplete femoral head are more consistent with limited hip motion movement. Thus, it is likely that *Microraptor* typically had its legs at various angles underneath its body during flight, as in some modern vultures [119].

#### Tibiotarsus: femur ratio and ecology

Xu et al. [17] previously described a dominant arboreal ecology for *Microraptor* based on their observations of the integument of IVPP V13352. Previously it has been suggested by Gatesy [94] that the tibiotarsus: femur ratio differs between non-avialan theropods (e.g. carnosaurs, ceratosaurs) and modern birds with non-avialan theropods showing a general tendency for a femur that is proportionally longer than modern birds in comparison to the tibiotarsus. This is due to differences in the positioning of the femur which is more perpendicularly oriented to the ground reaction force during the stride in running modern birds compared to non-avialan theropods [94]. In the same study, they found that ground-dwelling birds have a femur length of less than 80% of the tibiotarsus length. Here, we find that the tibiotarsus: femur ratio alone does not seem to yield ecologically relevant information for *Microraptor*. STM 6–86 displays a ratio at 1.48. Specimen LPM-B00169 of *Anchiornis* has a ratio of 1.6, whilst the early-diverging dromaeosaurid *Mahakala* has a ratio of 1.39 and the early-diverging troodontids *Mei long* and *Jinfengopteryx* have ratios of 1.32 and 1.43 respectively. Modern birds with a dominant cursorial behaviour such as the roadrunner (*Geococcyx*) or tinamou (*Eudromia*) have similar ratios of 1.54 and 1.41 respectively (using data of Jones et al. [120]). However, if we extend the sample of modern birds with data from Gatesy and Middleton [121], we observe that many other modern birds with various ecologies have similar ratios such as the arboreal Toco Toucan (*Ramphastus*) with a 1.51 ratio or the perching Belted Kingfisher (*Megaceryle*) with two specimens showing two different ratios: 1.39 and 1.48. This shows that this ratio varies a lot between and within modern bird species, which suggests that this ratio used alone is not ideal for uncovering ecological information in living and fossil paravians.

#### Hind limb: trunk ratio and phylogeny

Hind limb: trunk ratio has been suggested to be influenced by both phylogeny and size by Christiansen and Bonde [93] where they say that smaller theropods could have possessed proportionally longer limbs than larger forms. *Microraptor* does not seem to be an exception to this rule, so the high ratio has a potential phylogenetic explanation rather than an ecological one.

#### Ontogenetic status of study specimens and its implications

Our osteohistological observations suggest *Microraptor* D-2842 was approximately two years old when it died. This is consistent with the single resting line in the inner periosteal cortex separating the largely resorbed inner zone and relatively thick deposition of the consecutive zone. The animal was still actively growing at the time of death, although growth had already slowed down as exemplified by a significant drop in vascular density and randomness in the osteocyte distribution of the femur. Furthermore, the presence of the inner circumferential layer in both bones and the initial occurrence of the bone remodelling seen in the femur may constrain estimation of the specimen’s ontogenetic age as approaching a late stage of its juvenile period.

A comparison of osteohistology in *Microraptor* D-2842 suggests that microscopic signs of maturity have begun to develop at the end of the second recorded period which lasted most likely a year. For example, histological indicators were proposed to be more accurate in indicating adulthood in dromaeosaurids than their non-histological indicators [31]. We cannot rule out the possibility that this animal may well have been older and an earlier growth mark was removed by extensive resorption before the first endosteal bone occurred. This, however, is less likely when the studied specimen of *Microraptor* is compared with other dromaeosaurids and non-dromaeosaurid paravians of similar or smaller size as seen in Han et al. [28], Poust et al. [31], Prondvai et al. [95] and Cau et al. [122].

If we consider specimen D-2842 to be ~ 2 years old with a femur and tibia measuring 86 mm and 118 mm respectively, we can expect 8 specimens in our sample to be younger (BMHC PH881 and IVPP V12330, V13320, STM 5–75, 5–93, 5-150, 5-172 and 5-221) and 5 specimens to be older (IVPP V13352 and STM 5–5, 5–9, 5-142 and 6–86). However, specimen STM 6–62 shows a longer femur than D-2842 (97.73 mm compared to 86 mm) but has a shorter tibia than D-2842 (111.65 mm compared to 118 mm) making its relative ages difficult to determine. Specimen STM 6–62 suggests a degree of complexity in the growth of *Microraptor* bones with a tibia and femur that probably grow at different rates. If we consider that these bones can still grow at a late juvenile stage, this contrasts with modern birds that do not show longitudinal growth of their limb bones after the chick stage [123]. The fact that most specimens are younger than 2 years old means that our sample has many specimens that are not mature. However, it is still highly unknown whether some specimens are juveniles or adults in the fossil record of dinosaurs [124] and some species appear to be sexually mature before having a fully grown skeleton such as big theropods [125, 126]. In the same way, we observe that some modern birds (e.g. herons and cormorants) show that outer circumferential layer (OCL) growth as well as lamellar bone formation are not triggered by fledging or sexual maturity [127]. This shows that size remains a limited means to judge whether *Microraptor* was sexually mature enough or not. In the same way, the size of feathers seems to be dissociated with the growth of the legs with long feathers on small and potentially young *Microraptor* specimens such as BMHC PH881 and STM 5–75. We know that some modern birds are sexually mature and are successful breeders when they display fully grown feathers [128]. It could be similar for *Microraptor* with specimens of different sizes showing well-developed feathers (e.g. BMNHC PH881, STM 6–86) as observed on the early-diverging pygostylian *Confuciusornis sanctus* for whom specimens of different sizes display long rectrices [129]. For *Microraptor*, this hypothesis needs to be tested in the future with more specimens comprising well-preserved feathering. If we compare *Microraptor* with modern birds such as pelicans, we observe that these birds show some variation of size in the same species (*Pelecanus occidentalis*) with specimens being up to twice the size of others at the same growth stage [130]. This observation shows that we need to be cautious and that more studies on histology are required to determine the age of *Microraptor* specimens in the future in order to assess ontogeny.

The growth pattern of *Microraptor* D-2842 has been compared to other early paravians (see Additional file 1: Table S9). In *Microraptor* D-2842 the pattern involves 1 annulus/line of arrested growth (LAG), some fibro-lamellar bone (FLB) as well as an inner circumferential layer (ICL), which is almost identical to the closely related and much larger microraptorines *Changyuraptor* HG-B016 and *Sinornithosaurus* D-2140, indicating that *Microraptor* might have reached maturity comparatively faster. This trend is also exemplified by the more similar-sized microraptorine *Wulong* D-2933 that lacks any resting line where there is a significant decrease of vascular density towards the exterior of the tibia [31]. For example, the controversial specimen YFGP-T5198 sometimes assigned to the early-diverging paravian *Aurornis xui* [95, 131] or to *Anchiornis* [66] reached adulthood with a single growth interruption [95] and the early-diverging non-avian avialan *Jeholornis* (STM 2–51) with three growth cessations [132] at the size of the late juvenile *Microraptor* D-284.

No growth interruptions are seen in smaller early-diverging paravians: juvenile *Eosinopteryx* specimen YFGP-T5197, sub-adult *Anchiornis* specimen YFGP-T5199 and *Serikornis sungeri* PMOL-AB002000 [95]. On the contrary, numerous resting lines are found in later-diverging dromaeosaurids. The subadult *Buitreraptor* MPCA-PV-598 is twice as large as *Microraptor* D-2842 and considerably larger adults of *Dakotaraptor* (PBMNH.P.10.113.T, PBMNH.P.10.115.T) document variable patterns of decelerated maturation during the evolution of dromaeosaurids in the Late Cretaceous.

These observations provide valuable information that will help implement future work highlighting an evolutionary acceleration of hind limb growth in small-bodied paravian theropods, including early-diverging microraptorine dromaeosaurids.

#### Locomotor implications of the femoral accessory trochanteric crest

Situated at the base of the lesser trochanter, the femoral accessory trochanteric crest implies constraints on muscles of the *internus 2* group, which is associated with a hind limb: trunk ratio closer to that of modern running birds. This suggests an important locomotor role of the hind limbs for both flying and walking. The fact that these muscles were developed could also have allowed *Microraptor* to utilise powerful forward movements to attack prey, similar to modern birds of prey that use all of their extended toes to hit their victim from above [133]. Previously described pedal ungual morphology and pedal soft tissues are also consistent with a pinning role for digit II [2] that seems to imply a strategy to avoid injuring its legs during impacts with prey. This is correlated with the morphology of the forewing described by Grosmougin et al., (in this Collection) that shows similarities with modern falcons such as a similar wing shape as well as strongly developed flexor/stabiliser muscles that are inferred from its biceps tuberosity. These features displayed by *Microraptor* and some extant Falconiformes allow them to rapidly change position and it increases their velocity during the stoop to catch prey, a behaviour described by Bribiesca-Contreras et al. [134].

#### Pedal claw analysis and inferred behaviour

The claws of *Microraptor* have been previously described as potentially correlated with arboreal and terrestrial habitats [135–137] as well as having a role in hunt strategy [2]. The outer angle of the pedal unguals varies significantly and seems to be more consistent with the claw sheaths. Angles vary between ~ 110° and ~ 130° in IVPP V12330 and between ~ 130° and ~ 160° in specimens STM 5–75 and 5-172. It was not possible to correlate these results to other data such as body length and body mass. According to data and discussion from Feduccia [136], claw angles imply that specimens IVPP V12330 (~ 110–130°) and STM 6–62 (~ 110°) of *Microraptor* would have been similar to perching birds and specimen IVPP V13352 (~ 130–150°) as well as specimens STM 5–75 (~ 150–160°), 5–93 (~ 150°) and 5-172 (~ 140–160°) would have been more adapted to climbing tree trunks. We also found disparity in the pedal claw angle of STM 5–75 (~ 80°, ~ 120° and ~ 150°), which is in line with the analysis of Cobb and Sellers [138] that found two different claw morphologies in specimen CAGS 20-8-001 of *Microraptor* ( with evidence of perching on the right foot and predatory adaptation on the left foot. Note that measurements made by [136] on specimen CAGS 20-8-001 were from the left and right pedal digits III that do not preserve the claw sheath with a difference of 13° observed only between their inner angles: 92° for the left and 79° for the right. We also measured angles on this specimen and observed low inner angles compared to the previous study with ~ 45° on the right and ~ 55° on the left pedal. Concerning the outer angle, we observe higher values but much closer to those of the inner angle of the previous study and they show a ~ 20° difference with ~ 75° on the right and ~ 95° on the left (92° and 90° in Cobb and Sellers [138]). Our measurements seem to suggest a mistake in the original measurements of Cobb and Sellers [138] but we still recover a difference between the right and the left digit III pedal claws. We also found in specimen IVPP V12330, a pedal claw angle (without the claw sheath) varying from ~ 50–60° on the left foot to ~ 90° on the right one. This suggests that our data for STM 5–75, CAGS-20-8-001 as well as IVPP V12330 shows that pedal claw angle can vary in the same specimen so the ecological significance of claw angle should be considered with caution. Only digit II slightly differs from the other pedal digits if we only consider measurements without the pedal claw sheath in specimen IVPP V13352 as well as specimens STM 5–75, 5–93, 5-109 and 6–86. However, digit II does not exhibit a sufficient difference in specimen STM 5-172 (see Additional file 1: Table S8). The study of Hedrick et al. [139] demonstrated with geometric morphometric and traditional measurement data for modern birds that there is important intraspecific variation among different orders (i.e., Accipitriformes, Tinamiformes, Procellariiformes and Galliformes). This implies that we should use a large sample of specimens of the same species and combine these data with other information where possible. Although we observe a difference between the pedal claw of digit II and those of the other digits (without the sheath), with the data available it is currently not possible to describe an explicit role of the pedal claws of *Microraptor* as they do not show clear specialisation to arboreal or terrestrial lifestyles. Finally, if we consider digit I, we observe that it is not positioned as a hallux as previously described [2] and as in *Confuciusornis* [27, 91]. This feature is not consistent with a grasping role for the foot of *Microraptor*.

## Conclusions

This study sheds light on the morphology and function of *Microraptor* hind limbs using feather, soft tissue and bone data from well-preserved new and existing specimens. Feathers found on the legs of *Microraptor* illustrate an ancestral form that differs greatly from modern birds. This means that to explore the function of such feathery legs, many comparisons with fossil and modern examples are required. Feather data combined with soft tissues and skeletal characteristics demonstrate how complex the legs of *Microraptor* are. New features are described here for the first time such as asymmetrically vaned long metatarsal coverts linked to the metatarsus as well as rachises with different angles along the leg. These new data were interpreted alongside previously described elements such as long metatarsal remiges with potential aerodynamic advantages and the femoral accessory crest. Our updated hindwing reconstruction does not contradict the current prevailing view that the hindwing was held mostly vertically or slightly abducted during flight. Further evaluation of the detailed aerodynamic contribution of the hindwings to the flight performance of *Microraptor* will require additional modelling work that is outside the scope of this study. With our revised hindwing reconstruction and our proposal of its folding capability, *Microraptor* appears capable of both cursorial and arboreal activities. The pedal claw angles of *Microraptor* are found to have a wider variation than previously appreciated. This potentially points to a broader scope of usage; however, this remains tentative until further work can be conducted integrating additional lines of evidence. However, this animal certainly had good hunting skills reminiscent of some modern raptors and was likely to have used its enlarged digit II claw to pin prey. Building on previous studies, our work allows *Microraptor* to become one of best known early paravians with elongated hind limb feathers. This study makes progress in understanding the unique features found in early paravians which promise many more insights as other taxa are described in more detail.

Although this study samples the largest number of *Microraptor* specimens to this date and brings a wealth of new information, it also highlights many aspects that remain unknown. Thus, we should still consider more specimens moving forward as this will help to better assess ontogenetic and intrageneric variation as well as to provide additional support for the hypotheses proposed in this article.

## Electronic supplementary material

Below is the link to the electronic supplementary material.

Supplementary Material 1. Additional file 1 comprises MS Excel spreadsheets with all the numeric data as well as data used to compare fossil and modern specimens described and discussed in this article.

Supplementary Material 2. Additional file 2 comprises a MS Word document with all the supplementary figures with the preservation of the specimens of *Microraptor* described and discussed in this article.

## Data Availability

The datasets supporting the conclusions of this article are included within the article (and its additional files).

## Abbreviations

BMNHC: Beijing Museum of Natural History, Beijing, China
CAGS: Chinese Academy of Geological Sciences, Beijing, China
D & DNHM: Dalian Natural History Museum, Dalian, Liaoning, China
HG: Paleontological Center, Bohai University, Jinzhou, Liaoning, China
IVPP: Institute of Vertebrate Paleontology and Paleoanthropology, Beijing, China
LHV: Department of Land and Resources of Liaoning Province, Liaoning, China
LPM: Liaoning Paleontological Museum of Chaoyang National Geopark, Chaoyang, Liaoning, China
MPCA: Museo Provincial de Ciencias Naturales, General Roca, Río Negro, Argentina
PBMNH: The Palm Beach Museum of Natural History, Wellington, Florida, USA
PMOL: Paleontological Museum of Liaoning, Shenyang, Liaoning, China
STM: Shandong Tianyu Museum of Nature, Pingyi, Shandong, China
YFGP: Yixian Fossil and Geology Park, Yixian, Liaoning, China.5
