# Supplementary material for: New information on the Hind limb feathering, soft tissues and skeleton of Microraptor (Theropoda: Dromaeosauridae)
Source: BMC Ecol Evol. 2025 Apr 24;25:37. doi: 10.1186/s12862-025-02372-0 (PMC12020036; doi:10.1186/s12862-025-02372-0)
Supplement: Supplementary file 2 — Supplementary Material 2. Additional file 2 comprises a MS Word document with all the supplementary figures with the preservation of the specimens of Microraptor described and discussed in this article. [file 12862_2025_2372_MOESM2_ESM.docx]

**Supplementary material:**

**New information on the hind limb feathering, soft tissues and skeleton of *Microraptor* (Theropoda: Dromaeosauridae)**

Matthieu Chotard^1^, Xiaoli Wang^3,4,*^, Xiaoting Zheng^3,4,*^, Thomas G. Kaye^2^, Maxime Grosmougin^1^, Luke Barlow^1^, Martin Kundrát^5^, T. Alexander Dececchi^6^, Michael B. Habib^7^, Juned Zariwala^8^, Scott Hartman^9^, Xing Xu^10,11^ & Michael Pittman^1,*^

^1^School of Life Sciences, The Chinese University of Hong Kong, Shatin, Hong Kong SAR, China.

^2^Foundation for Scientific Advancement, Sierra Vista, Arizona, 85650, United States of America.

^3^Institute of Geology and Paleontology, Linyi University, Linyi City, Shandong, 276005, China.

^4^Shandong Tianyu Museum of Nature, Pingyi, Shandong, 273300, China.

^5^Center for Integrative Paleobiology, Technology and Innovation Park, Pavol Jozef Šafárik, University, SK-0401, Košice, Slovak Republic

^6^Division of Natural Sciences, Dakota State University, Madison, South Dakota, United States of America.

^7^David Geffen School of Medicine, University of California Los Angeles, Los Angeles, California, United States of America.

^8^School of Life and Environmental Sciences, College of Health and Sciences, University of Lincoln, Brayford Pool Campus, Lincoln, United Kingdom.

^9^Department of Integrative Biology, University of Wisconsin – Madison, Wisconsin, United States

^10^Centre for Vertebrate Evolutionary Biology, School of Life Sciences, Yunnan University, Chenggong, Kunming 650504, China.

^11^Key Laboratory of Vertebrate Evolution and Human Origins, Institute of Vertebrate Paleontology and Paleoanthropology, Chinese Academy of Sciences, Beijing 100044, China.

*Corresponding author: Michael Pittman ([mpittman@cuhk.edu.hk](mailto:mpittman@cuhk.edu.hkhotmail.com)), Wang Xiaoli ([wang_7355@163.com](mailto:wang_7355@163.com)), Xiaoting Zheng ([ty4291666@163.com](mailto:ty4291666@163.com))

**TABLE**

**Additional File 1:**

- **Table S1 Diagnostic characters in new *Microraptor* specimens**
- **Table S2 Feather pattern on hindlimbs of modern birds**
- **Table S3 Body mass estimation of *Microraptor* specimens**
- **Table S4 Measurements of *Microraptor* specimens hind limb feathers**
- **Table S5 Measurements of *Microraptor* specimens hind limb bones**
- **Table S6 Ratios of *Microraptor* specimens hind limb bones**
- **Table S7 Measurements and tibia/femur ratios of additional fossil Paraves**
- **Table S8 Measurements of Microraptor pedal claw angles**
- **Table S9 Details on the osteohistology of additional fossil Paraves**

**Additional File 2:**

- **Fig. S1 Hind limbs of *Microraptor* BMNHC PH881.**
- **Fig. S2 Hind limbs of *Microraptor* IVPP V13352.**
- **Fig. S3 Hind limbs of *Microraptor* STM 5-142.**
- **Fig. S4 Hind limbs of *Microraptor* STM 5-172.**
- **Fig. S5 Hind limbs of *Microraptor* IVPP V13320.**
- **Fig. S6 Hind limbs of *Microraptor* STM 5-9.**
- **Fig. S7 Hind limbs of *Microraptor* STM 5-4.**
- **Fig. S8 Hind limbs of *Microraptor* STM 5-221.**
- **Fig. S9 Hind limbs of *Microraptor* STM 5-109.**
- **Fig. S10 Hind limbs of *Microraptor* STM 5-150.**
- **Fig. S11 Hind limbs of *Microraptor* IVPP V12330.**
- **Fig. S12 Hind limbs of *Microraptor* STM 5-93.**


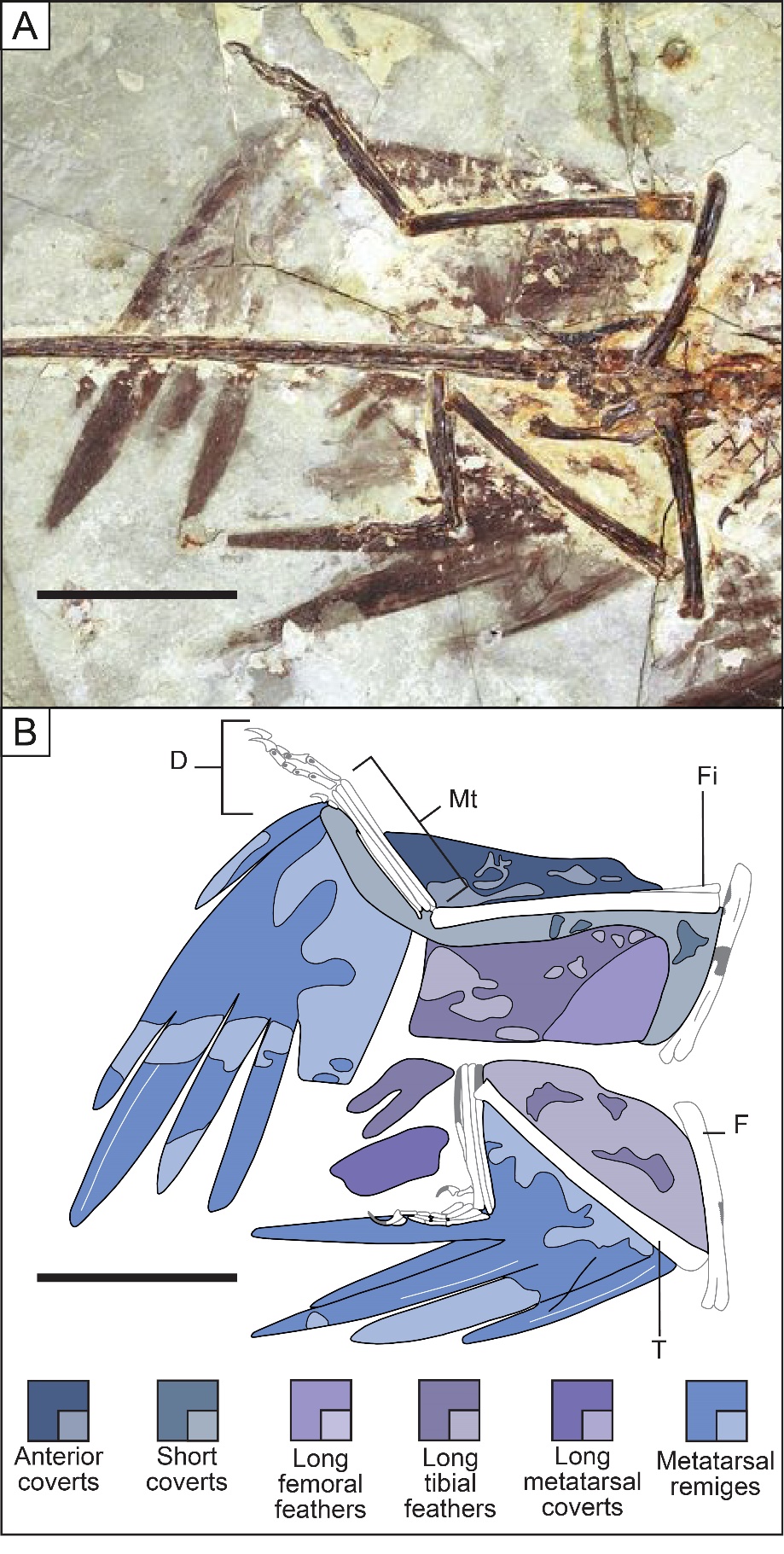


**Fig. S1 Hind limbs of Microraptor BMNHC PH881.** (A) WL image from the Fig. 1 in Li *et al.* (2012), (B) as an anatomical line drawing. D, left digits; F, left femur; Fi, left fibula; Mt, left metatarsus; T, right tibiotarsus. Scale bar is 50 mm.


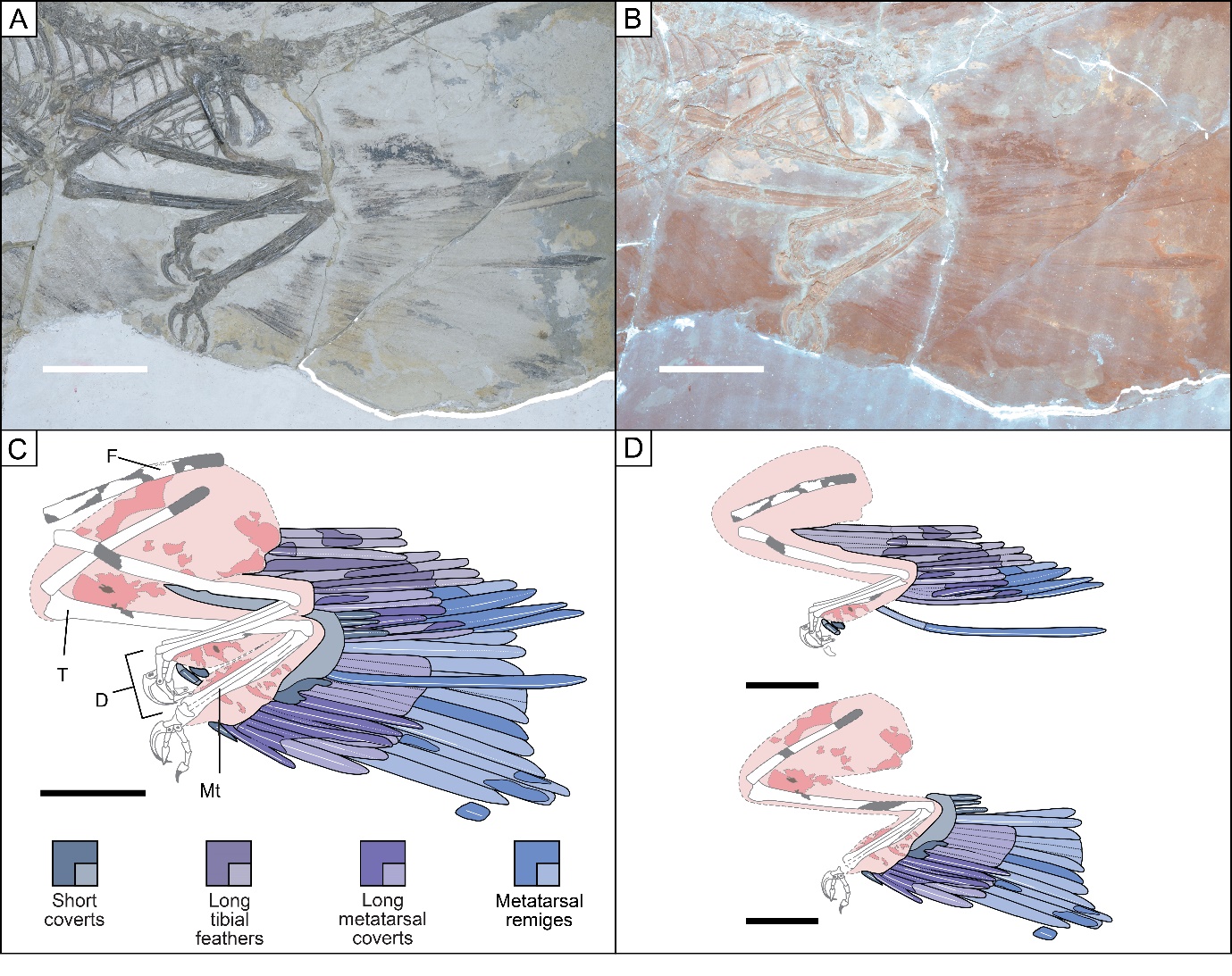


**Fig. S2 Hind limbs of *Microraptor* IVPP V13352.** (A) WL image of the slab with revised positioning of the slab portions, (B) LSF image of the slab with revised positioning of the slab portions, (C) as an anatomical line drawing of hindlimbs as observed on the fossil, (D) as an anatomical line drawing of hindlimbs separated. D, left digits; F, left femur; Mt, right metatarsus; T, right tibiotarsus. Scale bar is 50 mm.


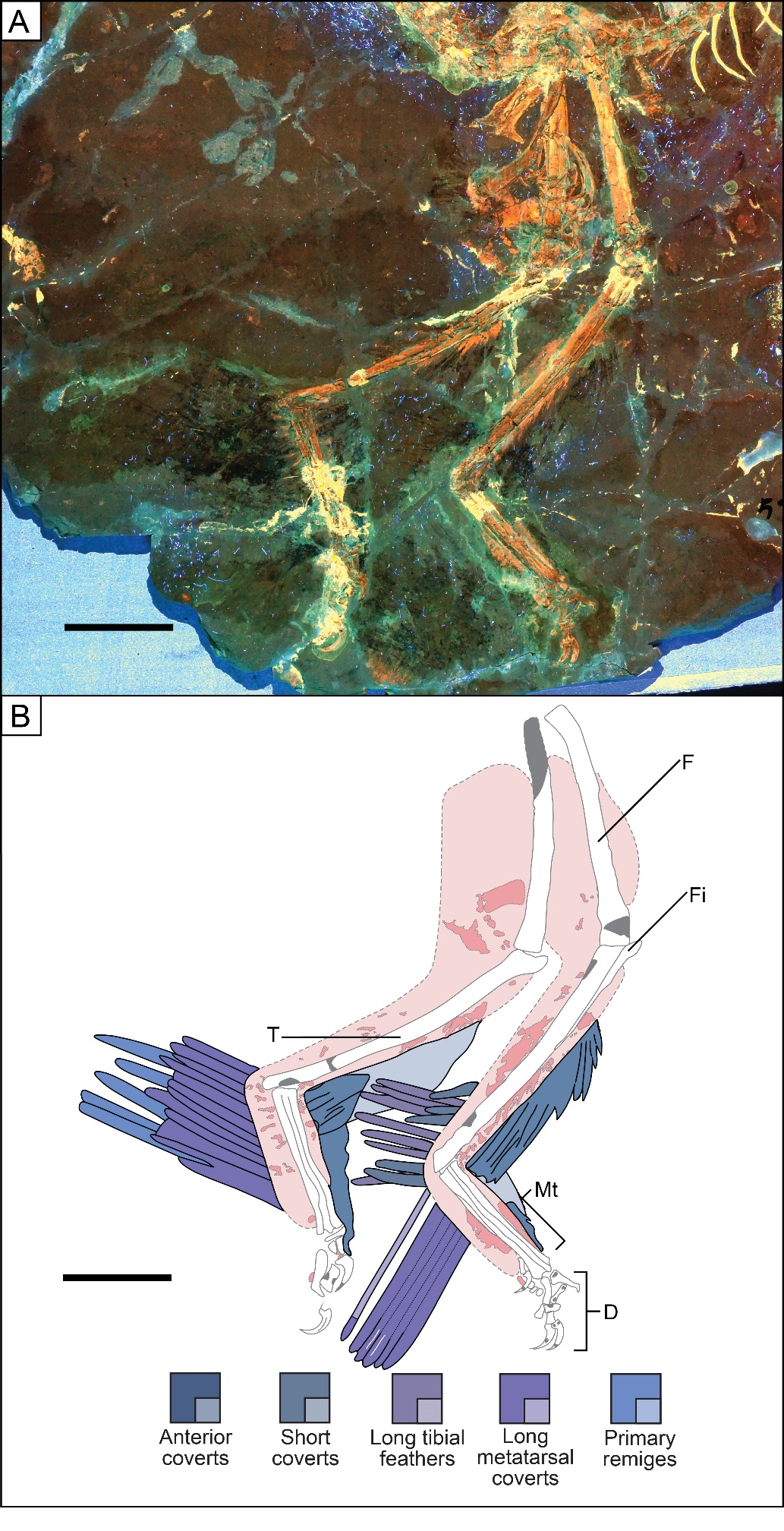


**Fig. S3 Hind limbs of *Microraptor* STM 5-142.** (A) LSF image of the slab, (B) as an anatomical line drawing. D, right digits; F, right femur; Fi, left fibula; Mt, right metatarsus; T, left tibiotarsus. Scale bar is 50 mm.


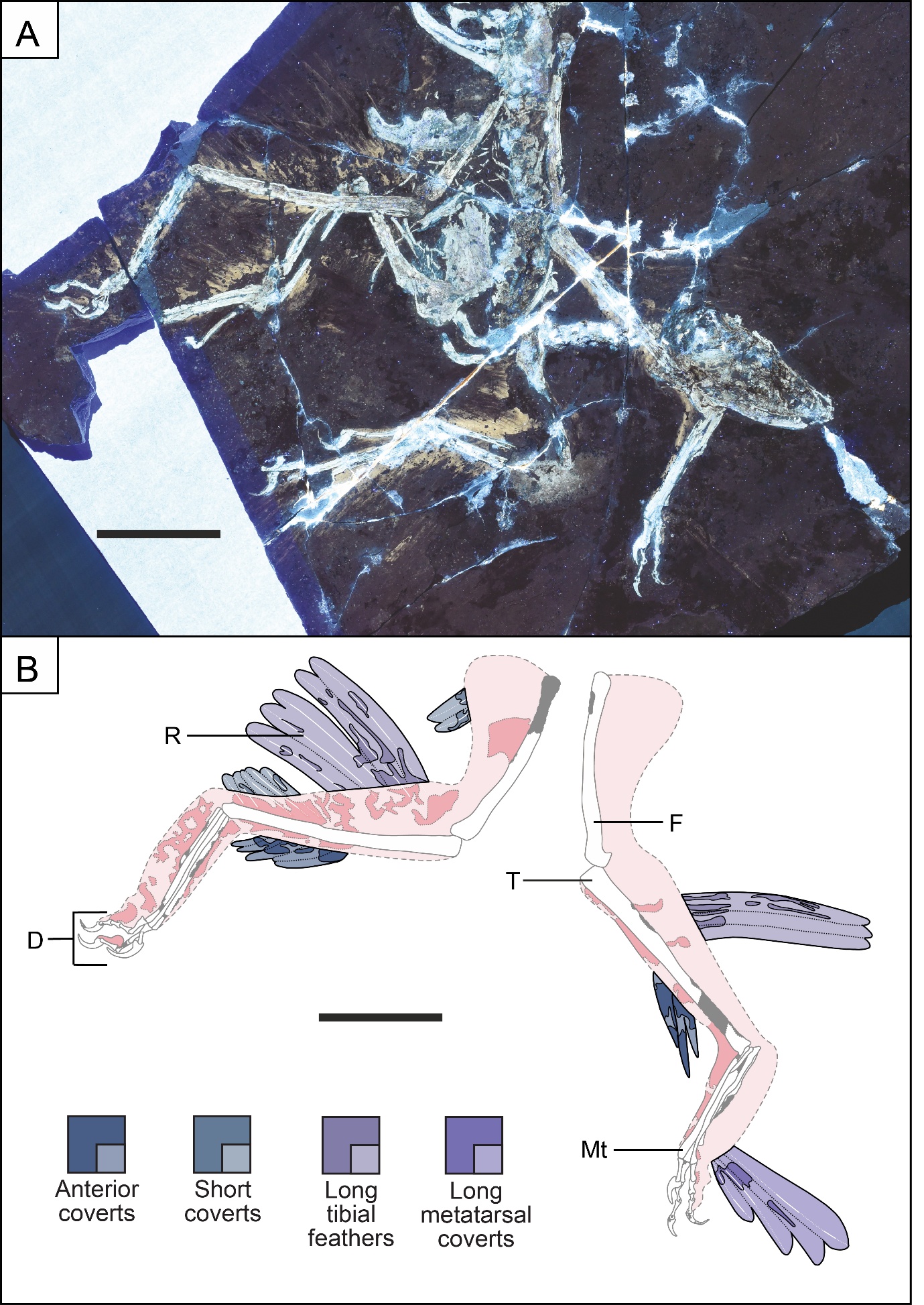


**Fig. S4 Hind limbs of *Microraptor* STM 5-172.** (A) LSF image of the slab, (B) as an anatomical line drawing. D, right digits; F, left femur; Mt, left metatarsus; R, preserved rachis; T, left tibiotarsus. Scale bar is 50 mm.


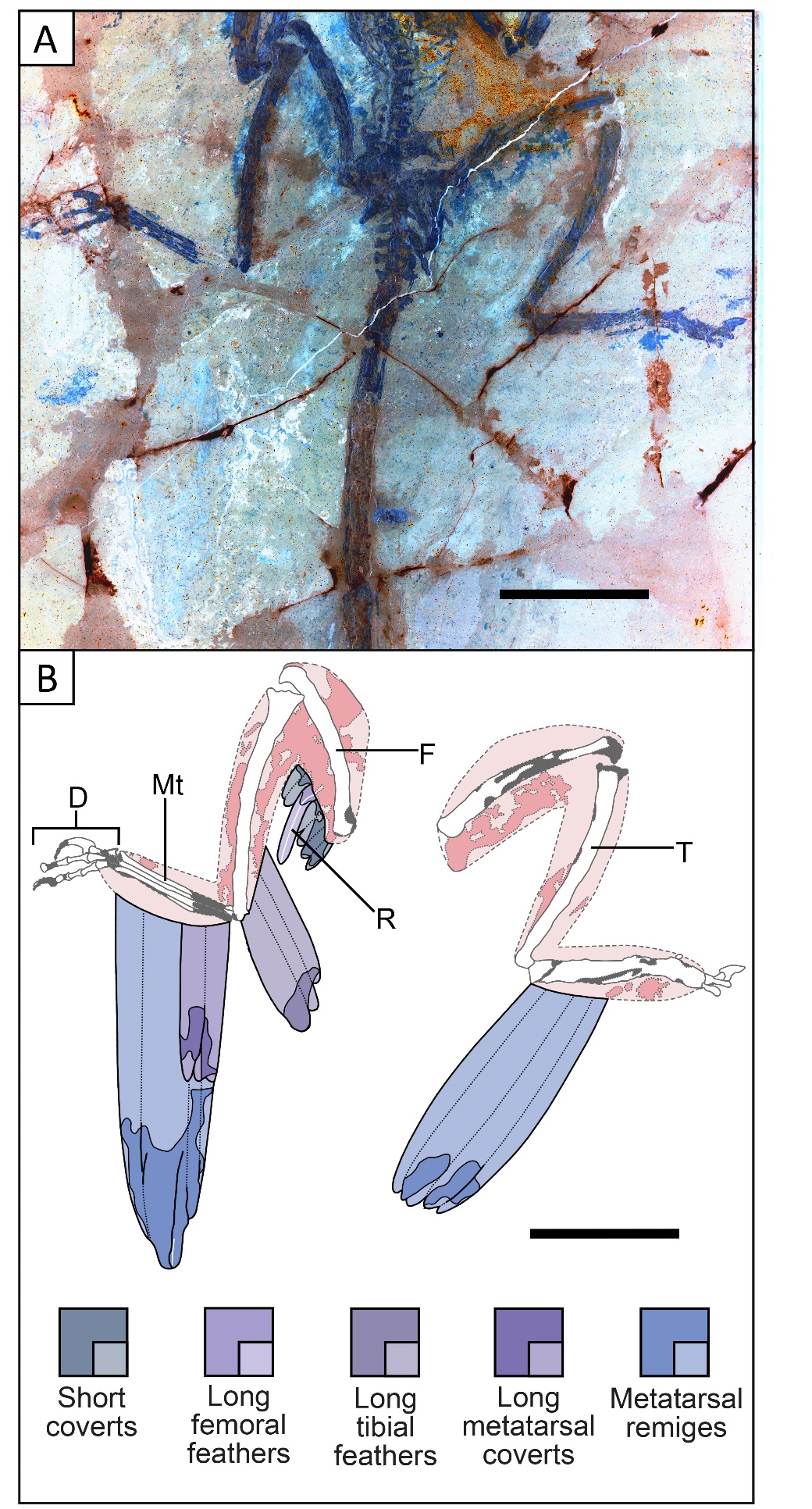


**Fig. S5 Hind limbs of *Microraptor* IVPP V13320.** (A) LSF image of the slab, (B) as an anatomical line drawing. D, left digits; F, left femur; Mt, left metatarsus; T, right tibiotarsus. Scale bar is 50 mm.


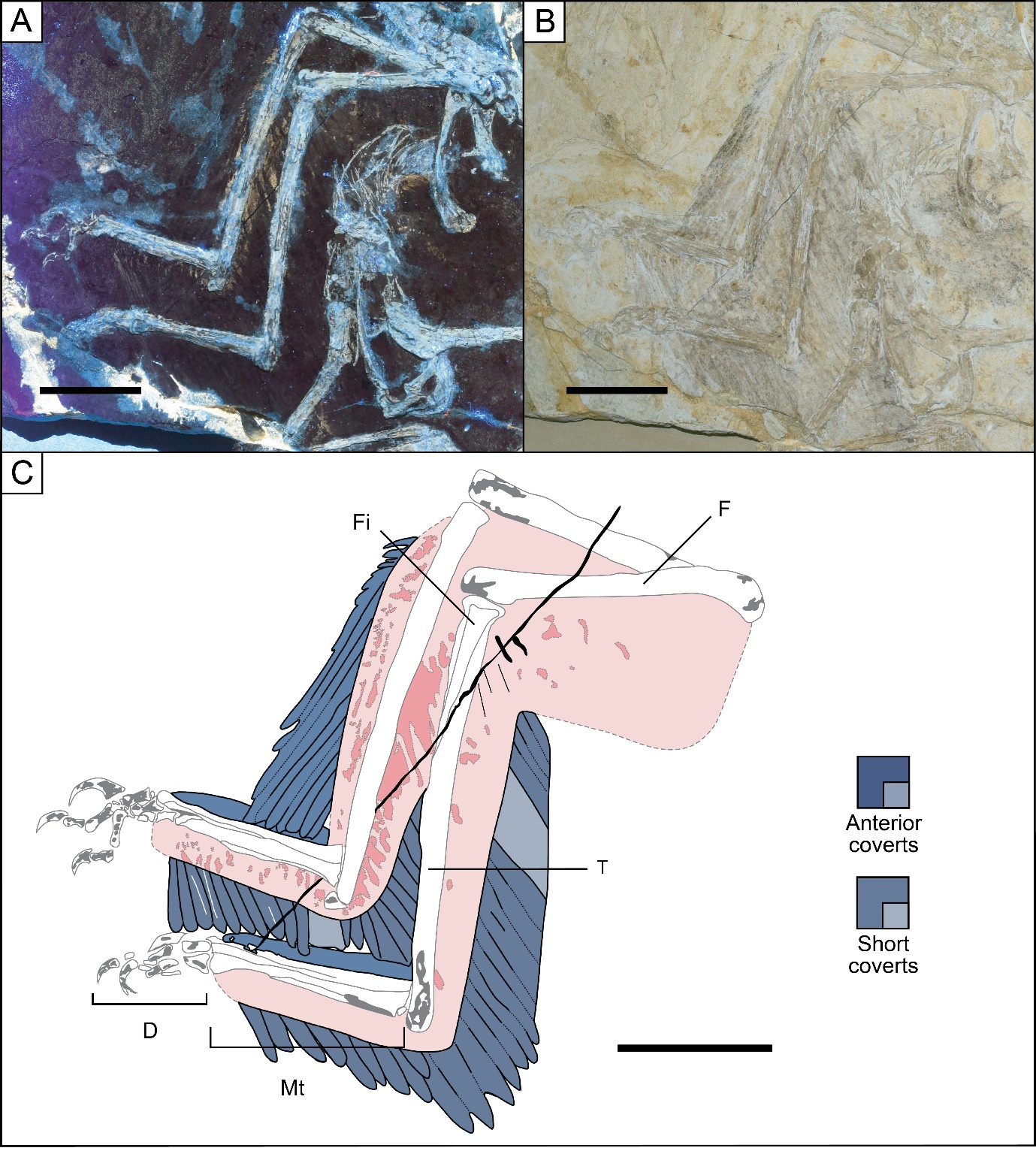


**Fig. S6 Hind limbs of *Microraptor* STM 5-9.** (A) LSF image of the slab, (B) WL image of the slab, (C) as an anatomical line drawing. D, left digits; F, left femur; Fi, left fibula; Mt, left metatarsus; T, left tibiotarsus. Scale bar is 50 mm.


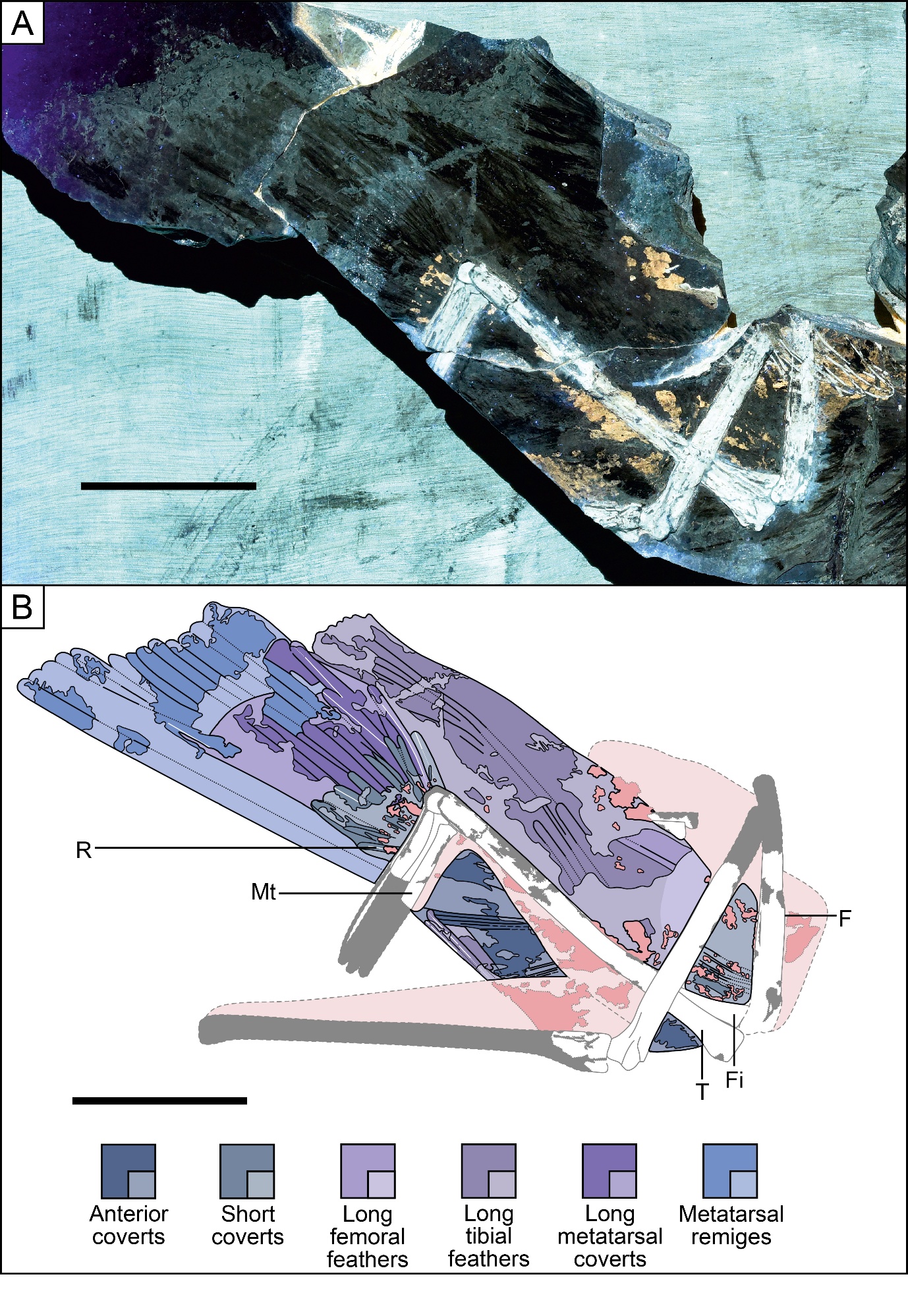


**Fig. S7 Hind limbs of *Microraptor* STM 5-4.** (A) LSF image of the slab, (B) as an anatomical line drawing. F, left femur; Fi, left fibula; Mt, left metatarsus; R, preserved rachis; T, left tibiotarsus. Scale bar is 50 mm.


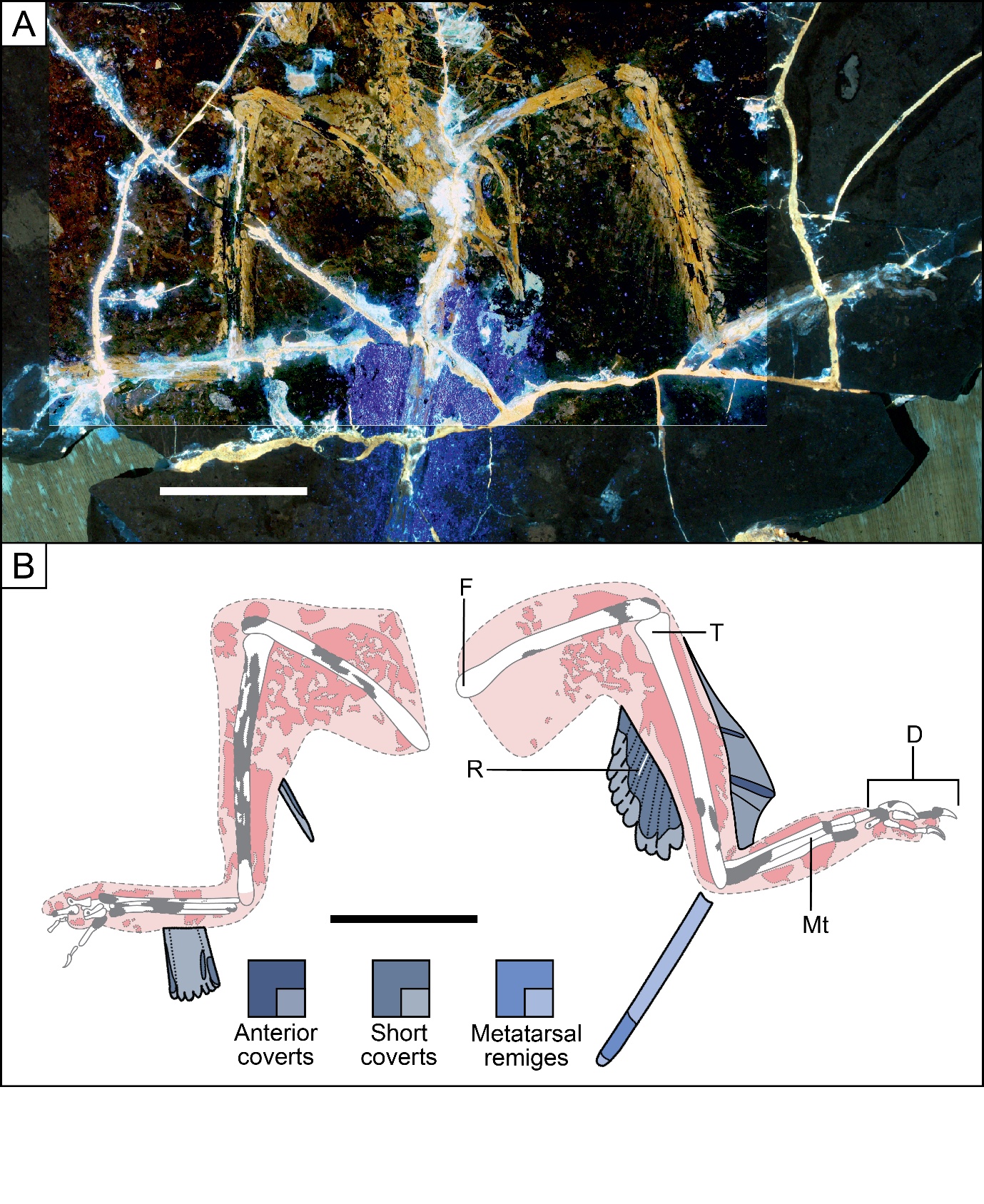


**Fig. S8 Hind limbs of *Microraptor* STM 5-221.** (A) LSF image of the slab, (B) as an anatomical line drawing. D, right digits; F, right femur; Mt, right metatarsus; R, preserved rachis; T, right tibiotarsus. Scale bar is 50 mm.


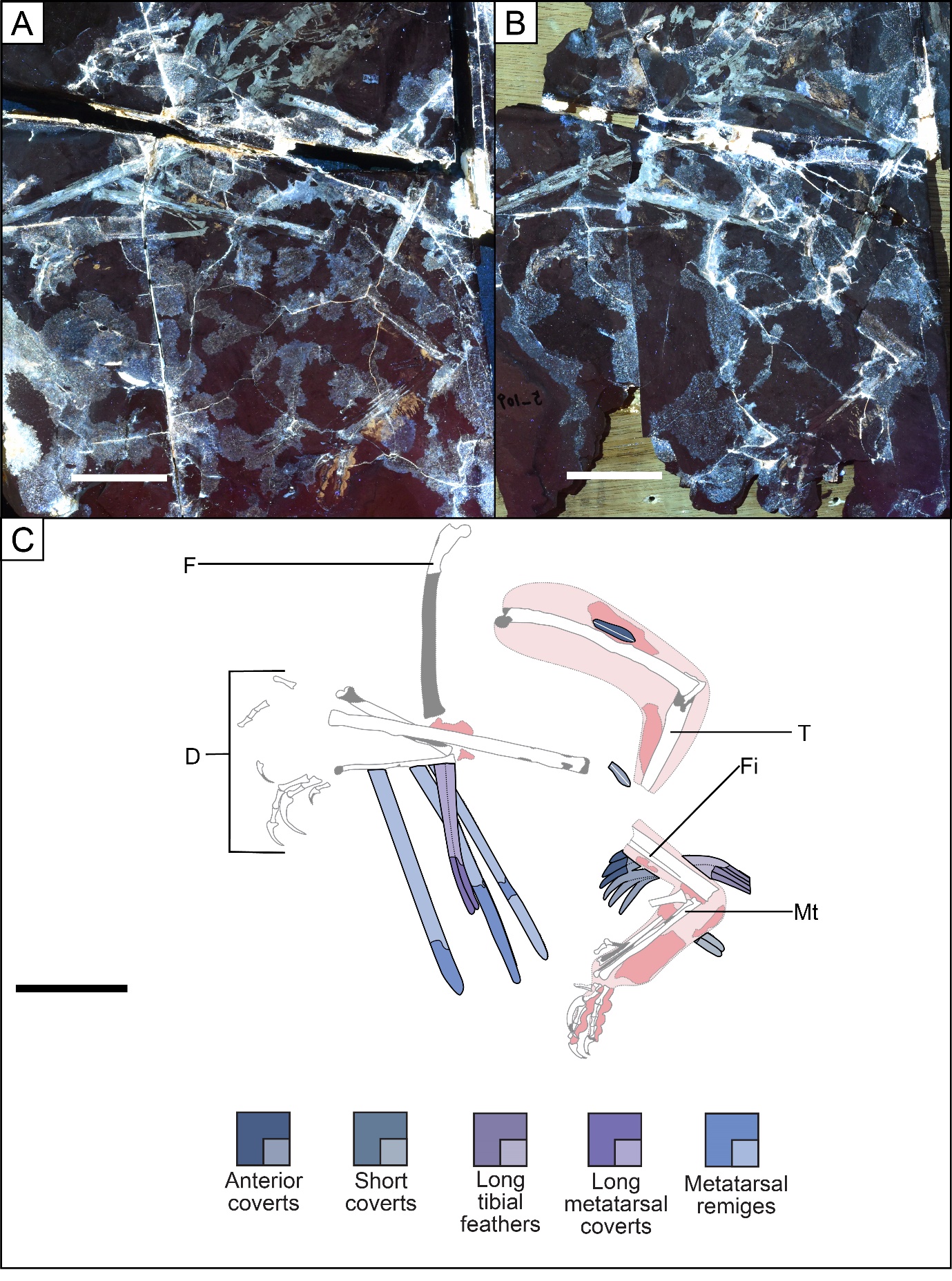


**Fig. S9 Hind limbs of *Microraptor* STM 5-109.** (A) LSF image of the slab, (B) LSF image of the counter-slab, (C) as an anatomical line drawing. D, right digits; F, right femur; Fi, left fibula; Mt, left metatarsus; T, left tibiotarsus. Scale bar is 50 mm.


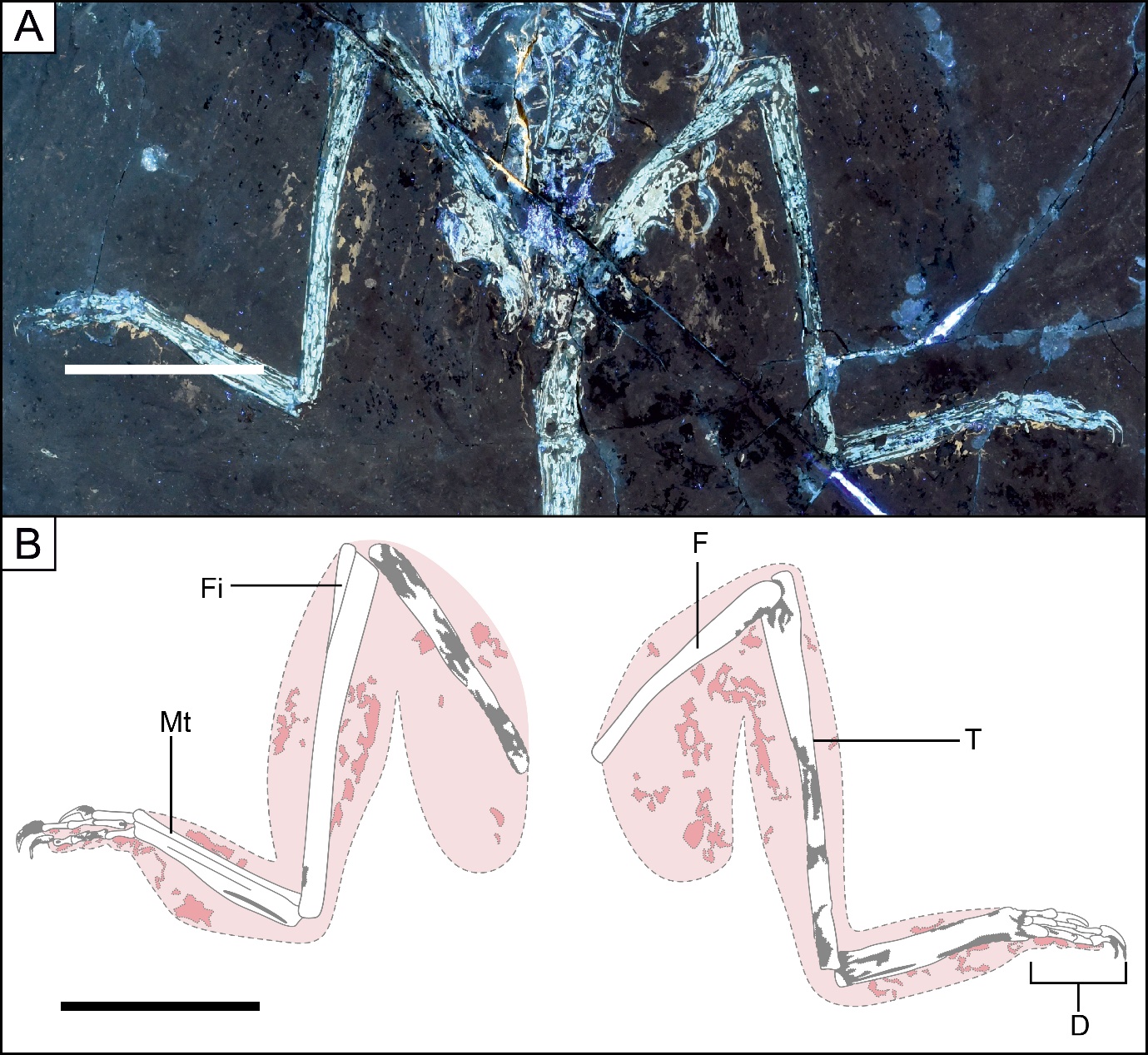


**Fig. S10 Hind limbs of *Microraptor* STM 5-150.** (A) LSF image of the slab, (B) as an anatomical line drawing. D, right digits; F, right femur; Fi, left fibula; Mt, left metatarsus; T, right tibiotarsus. Scale bar is 50 mm.


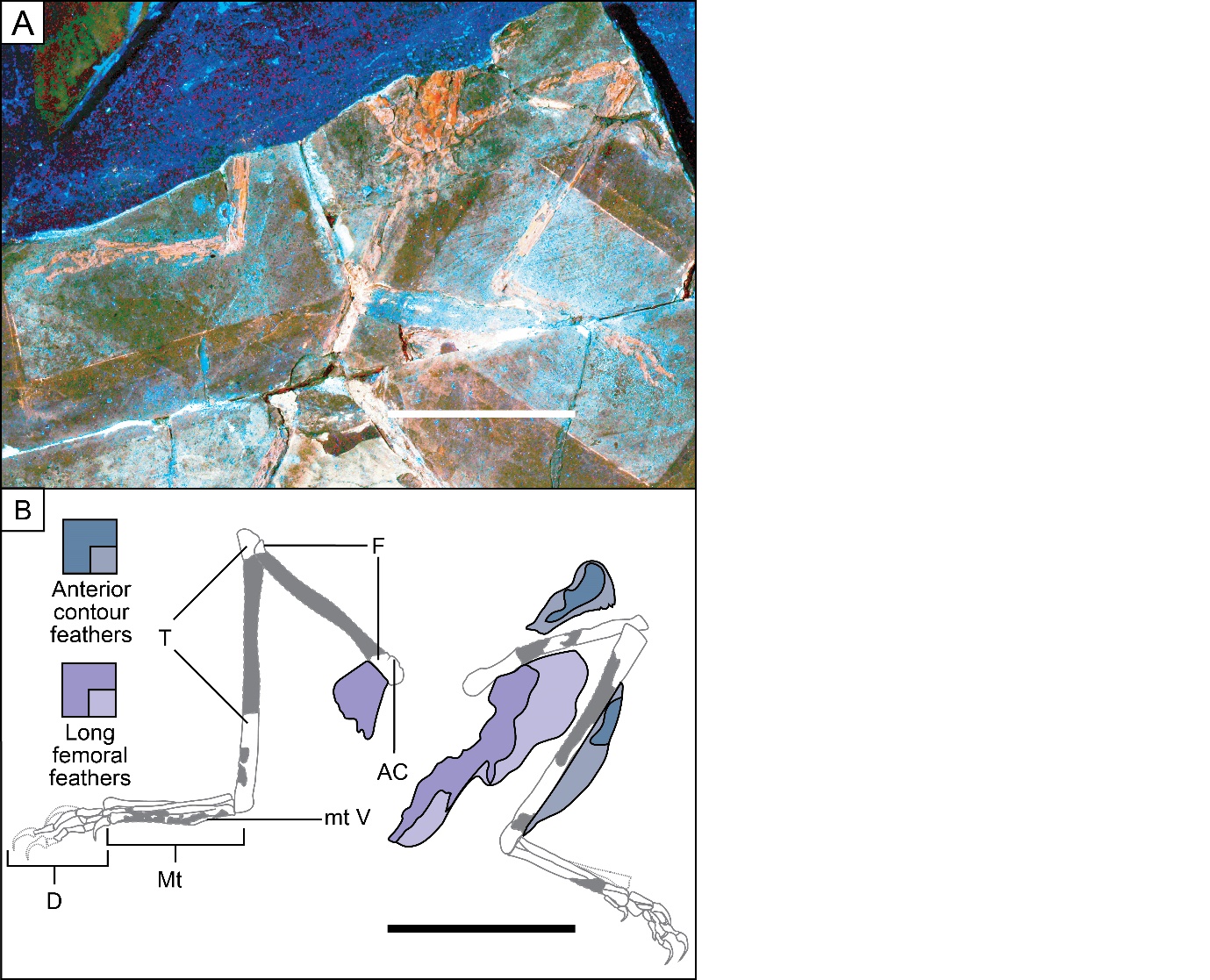


**Fig. S11 Hind limbs of *Microraptor* IVPP V12330.** (A) LSF image of the slab, (B) as an anatomical line drawing. AC, accessory crest; D, right digits; F, right femur; Mt, right metatarsus; mt V, right metatarsus V; T, right tibiotarsus. Scale bar is 50 mm.


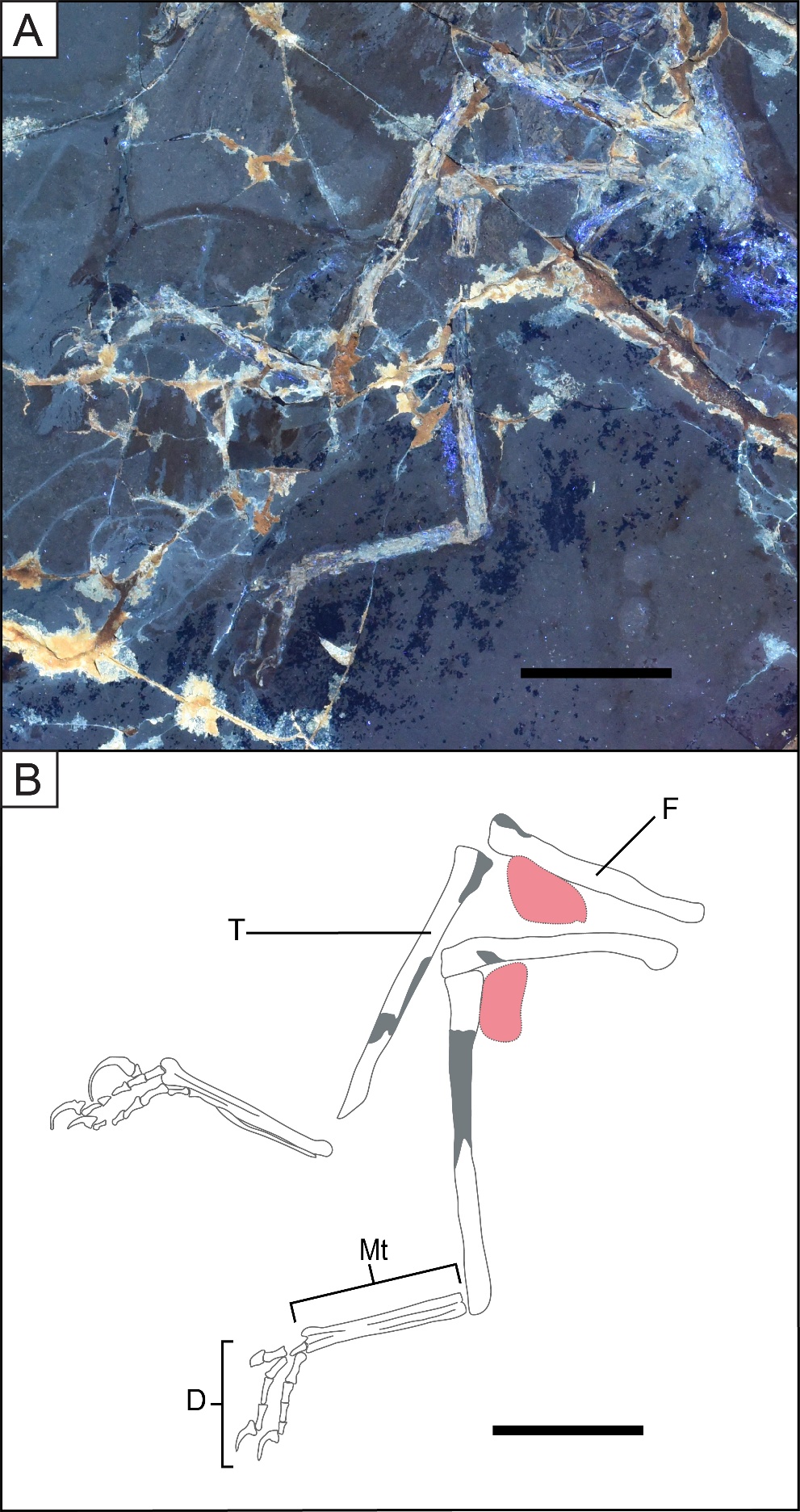


**Fig. S12 Hind limbs of *Microraptor* STM 5-93.** (A) LSF image of the slab, (B) as an anatomical line drawing. D, left digits; F, right femur; Mt, left metatarsus; T, right tibiotarsus. Scale bar is 50 mm.
